# AARS1‐Mediated RUNX3 K193 Lactylation Enhances the Activation and Cytotoxicity of CD8^+^T Cells in Smoking‐Related Emphysema

**DOI:** 10.1002/advs.76949

**Published:** 2026-08-06

**Authors:** Ying Zhu, Jiaheng Lin, Yifan Li, Qiaoqiao Zhou, Min Yu, Cheng Cheng, Kang Chang, Dang Lin, Tao Bian, Yan Wu, Lu Lu, Haibo Xia, Qizhan Liu

**Affiliations:** ^1^ Center For Global Health The Key Laboratory of Modern Toxicology Ministry of Education School of Public Health Suzhou Institute for Advanced Study of Public Health Gusu School Nanjing Medical University Nanjing Jiangsu People's Republic of China; ^2^ Department of Respiratory and Critical Care Medicine The Affiliated Wuxi People's Hospital of Nanjing Medical University Wuxi Medical Center Nanjing Medical University Wuxi Jiangsu People's Republic of China; ^3^ Department of Respiratory and Critical Care Medicine The Affiliated Suzhou Hospital of Nanjing Medical University Suzhou Municipal Hospital Nanjing Medical University Suzhou Jiangsu People's Republic of China; ^4^ Animal Core Facility Jiangsu Animal Experimental Center of Medical & Pharmaceutical Research Nanjing Medical University Nanjing Jiangsu People's Republic of China

**Keywords:** AARS1, CD8^+^T cell activation, cytotoxicity, emphysema, lactylation, smoking

## Abstract

Emphysema is the primary pathological feature of chronic obstructive pulmonary disease (COPD). The accumulation of CD8^+^T cells induces epithelial cell damage, thereby impairing the pulmonary alveolar niche. In this study, we demonstrated that CD8^+^T cell activation was associated with COPD progression. Furthermore, our data indicated that AARS1‐mediated lactylation was involved in cigarette smoke extract (CSE)‐induced CD8^+^T cell activation. Mechanistically, cigarette smoke (CS) exposure upregulated AARS1 levels in CD8^+^T cells. Elevated AARS1 catalyzed the transfer of a lactyl group to RUNX3 at the K193 site, leading to the elevation of lactylation of RUNX3‐K193, and inhibited RUNX3 fusion with autophagosomes for degradation. Subsequent accumulation of RUNX3 promoted CD8^+^T cell activation and enhanced their cytotoxicity. Here we demonstrate, using a CD8^+^T cell‐lung organoid co‐culture model and an experimental emphysema model induced by CS, that treatment with the lactate inhibitor DCA and silencing of AARS1 abrogated CS‐induced CD8^+^T cell activation, which ameliorated emphysematous pathological changes in mouse lung tissue. Moreover, elevated lactate and AARS1 negatively correlated with lung function in human populations. Collectively, this study reveals that AARS1 drives CD8^+^T cell activation by mediating RUNX3 lactylation to inhibit its autolysosomal degradation, and targeting of AARS1 may provide new insights into the therapy of emphysema.

## Graphical abstract

Exposure to CS causes an elevation of AARS1 levels, which increases the levels of RUNX3 lactylation at the K193 site and increases protein levels of RUNX3 through inhibition of autolysosomal degradation. Elevated RUNX3 levels promote CD8^+^T cell activation and augment their cytotoxicity, which induces alveolar epithelial cell death and facilitates the development and progression of emphysema.

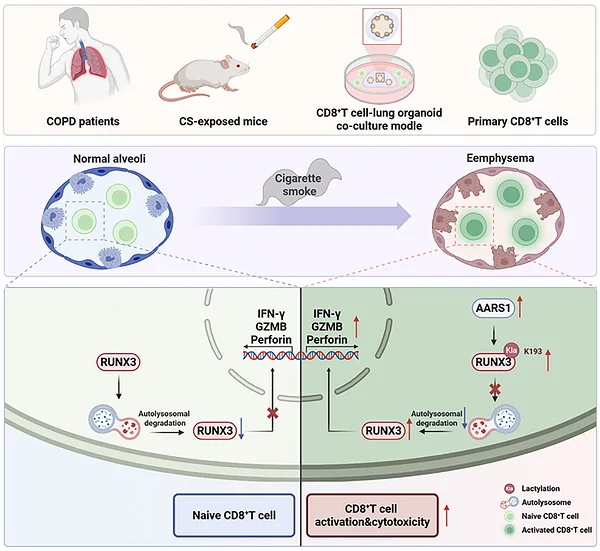

## 1 Introduction

Chronic obstructive pulmonary disease (COPD) manifests as persistent respiratory symptoms and airflow limitation [1]. COPD, the third leading cause of death globally, imposes a heavy public health burden worldwide [2]. China bears the largest COPD burden, with nearly 100 million adult patients and high mortality largely attributed to tobacco smoking and ambient air pollution [3]. Among its manifestations, emphysema represents the most severe pathological alteration in COPD, resulting from the interaction and cumulative effects of tobacco exposure, occupational pollutants, and other environmental risk factors along with genetic predisposition [4]. A histological feature driving emphysema pathology is alveolar epithelial cell death and depletion [5]. Moreover, alveolar epithelial cells have the capacity for repair and regeneration, which can repopulate damaged areas to restore alveolar structure and function [6]. This repair process is constrained by chronic immune infiltration; however, the underlying mechanisms remain unclear. Therefore, elucidating the biological mechanisms of COPD is important for developing effective therapeutic methods for the disease.

CD8^+^T cells, as core effector cells of the adaptive immune system, are rapidly activated into an effector state upon antigen recognition or exposure to environmental pollutants, thereby contributing to the pathogenesis of respiratory diseases [7]. Specifically, upon activation into cytotoxic T lymphocytes (CTLs), CD8^+^T cells rapidly proliferate and secrete large amounts of proinflammatory and cytotoxic factors such as Perforin, granzyme B (GZMB), and interferon‐gamma (IFN‐γ) to kill alveolar epithelial cells [8, 9]. Clinical reports show elevated total T lymphocyte counts in the lung parenchyma, peripheral airways, and central airways of COPD patients [5].

The transcription factor RUNX3 (runt‐related transcription factor 3) acts as a regulator of CD8^+^T cell differentiation and effector function [10]. It binds the promoters of Perforin, GZMB, and IFN‐γ to initiate the transcriptional program of cytotoxic CD8^+^T cells and to maintain their tissue‐resident phenotype in inflamed lungs [11]. RUNX3 deletion impairs CTL clonal expansion and suppresses the release of cytotoxic mediators, thereby alleviating pulmonary immune injury [12]. Despite its well‐established role in CD8^+^T cell immunity, few studies have addressed whether RUNX3‐mediated CD8^+^T cell overactivation drives smoke‐induced emphysema, and the upstream mechanisms underlying aberrant RUNX3 expression in COPD remain unclear.

Abnormal metabolism in immune cells influences immune cell function, as CD8^+^T cells are subject to metabolic reprogramming of aerobic glycolysis and fatty acid synthesis that influence their functional capacity [13]. Alterations in glycolysis and oxidative phosphorylation (OXPHOS) pathways in CD8^+^T cells exert differential regulatory effects on the cellular activation, lineage differentiation, and effector function of this immune subset [14]. However, the functional changes induced by altered cellular metabolism extend beyond traditional roles in bioenergetics and synthesis. Specific metabolites regulate immune cell activation and function by participating in post‐translational modifications [15]. Lactic acid acts as a substrate for the lactylation modification and thereby participates in diverse physiological and pathological processes, such as immune cell differentiation, tumor progression, and fibrotic tissue formation [16]. As a newly identified lactyltransferase, alanyl‐tRNA synthetase 1 (AARS1) bridges glycolytic lactate accumulation and protein lactylation, serving as a mediator linking metabolic reprogramming to epigenetic regulation [17]. Although AARS1 modulates innate immune responses and tumor progression, its potential role in adaptive CD8^+^T cell dysfunction during smoking‐induced pulmonary injury remains poorly defined. However, the impact of lactylation on regulation of CD8^+^T cell activation in smoking‐associated emphysema is largely uncharacterized.

When we applied single ‐ cell RNA sequencing (scRNA‐seq) analysis, the findings indicated that CD8^+^T cell activation was increased in the lungs of COPD patients. For CS‐exposed mice, CD8^+^T cell activation was involved in emphysema pathological changes. Mechanistically, exposure to cigarette smoke (CS) increased AARS1 levels in CD8^+^T cells, thereby mediating lactylation of RUNX3 at the K193 site to inhibit its autolysosomal degradation. Moreover, the increased levels of RUNX3 contribute to CD8^+^T cell activation and enhance its cytotoxicity, inducing alveolar epithelial cell death and contributing to the development and progression of emphysema. Thus, the present study reveals an interplay between lactate and CD8^+^T cell activation and demonstrates that _β_LNPs@AARS1‐siRNA represents a promising translational strategy for targeting CD8^+^T cells in smoking‐related emphysema.

## 2 Results

### 2.1 Elevation of CD8^+^T Cell Activation in Patients with COPD and in an Experimental Emphysema Model

To elucidate the cellular phenotypic characteristics of lungs of patients with COPD, we analyzed scRNA‐seq data from the GSE196638 dataset. Through dimensionality reduction and clustering analyses, 11 distinct cell lineages with lineage‐specific marker gene expression were identified from lung samples of 3 healthy controls and 3 COPD patients (Figure S1A–E). Notably, relative to healthy controls, the proportions of T cells in the lungs of COPD patients were elevated by up to 41% (Figure S1F–H). Uniform manifold approximation and projection (UMAP) analysis identified 4 clusters of T cells (Figure 1A; Figure S2A–D). Among these, the proportion of CD8^+^T cells in the lungs of COPD patients was elevated by 38% relative to that for healthy controls (Figure 1B; Figure S2E,F). Additionally, enrichment analyses based on the Kyoto Encyclopedia of Genes and Genomes (KEGG) and Gene Ontology (GO) databases demonstrated that the T cell receptor signaling pathway and the T cell activation signature were enriched in CD8^+^T cells derived from COPD patients (Figure 1C,D; Figure S3A,B). Pseudotime trajectory analysis of CD8^+^T cells demonstrated that naive CD8^+^T cells were clustered predominantly in healthy individuals, whereas activated CD8^+^T cells were enriched in COPD patients (Figure 1E,F; Figure S3C,D). We also collected lung tissues from healthy individuals and from COPD patients (Table S1). Immunofluorescence (IF) staining confirmed elevated numbers of CD8a^+^ cells in the lungs of COPD patients (Figure 1G). To validate the robustness of our findings, we analyzed scRNA‐seq data from the GSE173896 dataset; the results were consistent with those obtained from the GSE196638 dataset (Figure S4).

**FIGURE 1:**
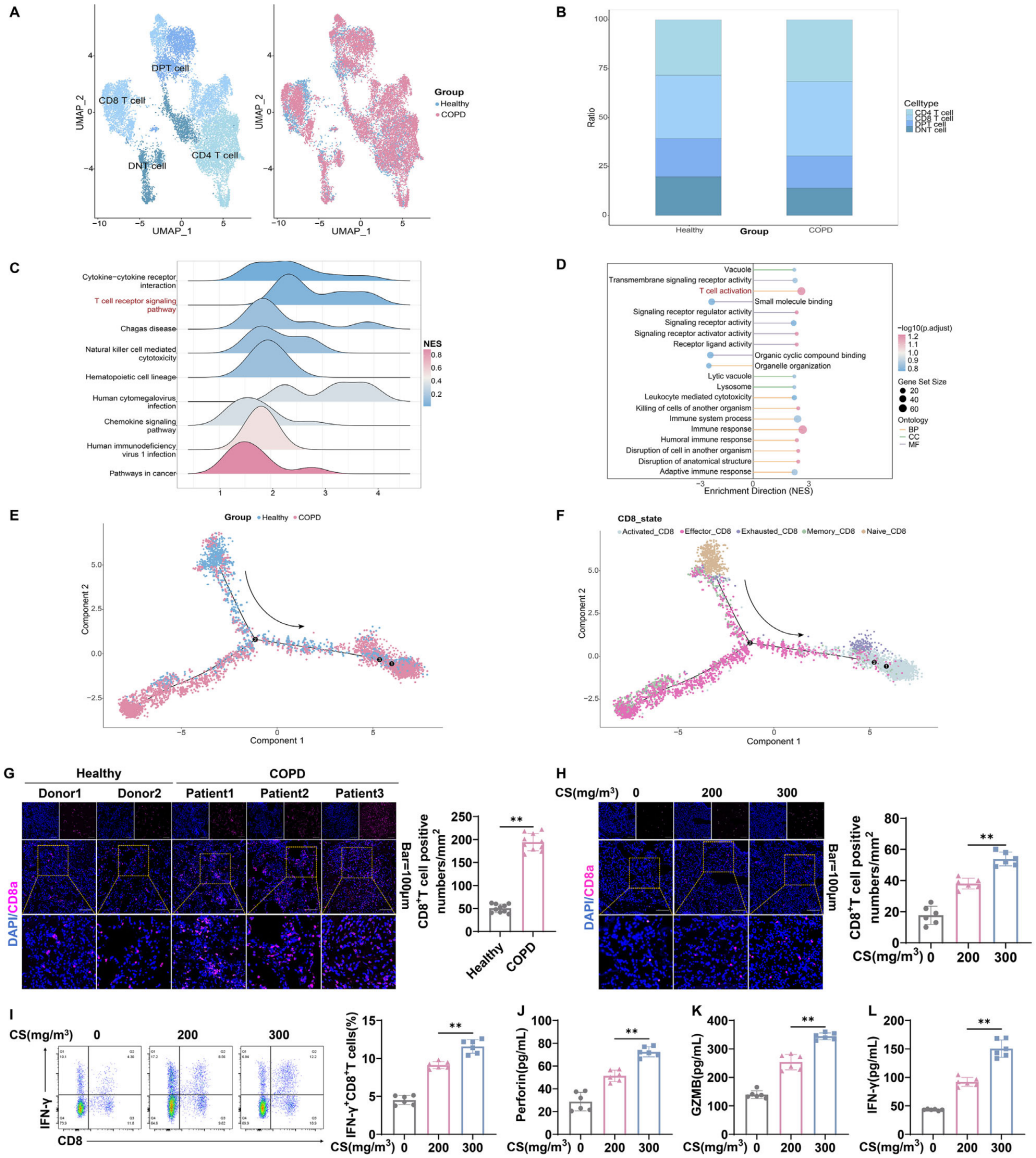
The activation of CD8^+^T cells is elevated in COPD patients and emphysema mouse models. Analysis of scRNAseq data from lungs of healthy individuals (*n* = 3) and COPD patients (*n* = 3) in the GSE196638 database. (A) UMAP analysis of T cells from healthy individuals and COPD patients. (B) Box plots showing the proportions of distinct cell clusters in T cells of healthy individuals and COPD patients. KEGG (C) and GO (D) enrichment analysis of dysregulated mRNAs from T cells of healthy individuals and COPD patients. (E) Pseudo‐time differentiation trajectories of CD8^+^T cells in healthy individuals and COPD patients. (F) Pseudo‐time differentiation trajectories of distinct cell clusters in CD8^+^T cells. Analysis of lungs and serum from healthy individuals and COPD patients. (G) Representative CD8a immunostaining images (left) and corresponding quantitative analysis (right) of lungs from healthy donors (*n* = 10, biological replicates) and COPD patients (*n* = 10, biological replicates) (scale bar, 100 µm). Six‐week‐old male BALB/c mice were exposed to cigarette smoke (CS) for 24 weeks prior to subsequent experiments, with total particulate matter (TPM) concentrations in smoke set at 0, 200, or 300 mg/m^3^. (H) Representative immunostaining images (left) and quantitative analysis (right) of CD8a in lungs from mice (*n* = 6, biological replicate) (scale bar, 100 µm). (I) Representative flow cytometry profiles (left) and quantitative analysis (right) of IFN‐γ^+^CD8^+^T cells isolated from mouse lungs (*n* = 6, biological replicate). Perforin (J), GZMB (K), and IFN‐γ (L) levels in serum of mice (*n* = 6, biological replicates) were quantified by ELISA. Data are presented as means ± SEM. ^**^ *p* < 0.01, by one‐way ANOVA followed by Bonferroni correction or unpaired Student's *t*‐test.

To assess CD8^+^T cell activation in emphysema, we established a CS‐induced mouse model of emphysema (Figure S5A–D). In lungs of CS‐exposed mice, levels of CD8a^+^ cells increased in a dose‐dependent manner (Figure 1H). Flow cytometry analysis showed that IFN‐γ^+^CD8^+^T cells were elevated in lungs of CS‐exposed mice (Figure 1I; Figure S5E). The levels of CD8^+^T cell activation‐related factors, Perforin, granzyme B (GZMB), and interferon‐γ (IFN‐γ), were upregulated in serum of CS‐exposed mice (Figure 1J‐L). Consistent with in vivo results, the proportions of IFN‐γ^+^CD8^+^T cells were elevated in CSE‐treated primary mouse CD8^+^T cells with elevations of Perforin, GZMB, and IFN‐γ (Figures S6A–H and S7). As IFN‐γ derived from cytotoxic CD8^+^T cells acts as a well‐established negative regulator of the canonical Wnt/β‐catenin pathway, activated CD8^+^T cells suppress Wnt signaling in alveolar epithelial cells by accelerating β‐catenin degradation, disrupting epithelial homeostasis, and promoting epithelial apoptosis [18, 19]. To further confirm that the impaired Wnt signaling and epithelial apoptotic remodeling were specifically caused by CD8^+^T cell activation rather than nonspecific CSE effects, we applied gradient anti‐CD3/CD28 stimulation to mimic graded TCR‐dependent CD8^+^T cell activation. Notably, TCR stimulation dose‐dependently increased the secretion of cytotoxic cytokines (Figure S8A–C). After co‐culture with CSE‐treated CD8^+^T cells, MLE‐12 cells showed lower expression of Wnt/β‐catenin pathway‐related proteins (Axin2 and β‐catenin) and of the anti‐apoptotic protein Bcl‐2, along with increased expression of the pro‐apoptotic protein Bax (Figure S6I,J). Consistently, identical phenotypic changes were recapitulated when MLE‐12 cells were co‐cultured with CD3/CD28‐activated CD8^+^T cells (Figure S8D). Collectively, these data demonstrate that the expansion of activated CD8^+^T cells may be involved in CS‐induced emphysema.

### 2.2 CSE Triggers Glycolytic Metabolic Reprogramming of CD8^+^T Cells

Lactate metabolic dynamics govern lysine lactylation of both histone and non‐histone proteins, and thereby modulate gene expression and protein activity in immune cells via glycolysis‐dependent signaling cascades [20]. In CD8^+^T cells, exposure to environmental stimuli triggers glycolytic metabolic reprogramming, which in turn leads to a buildup of lactate inside the cells. This lactate‐driven epigenetic process pushes CD8^+^T cells to shift from a resting state to an activated, functional state, and, in doing so, boosts their capacity to kill cells and secrete pro‐inflammatory signaling molecules [21]. Therefore, we performed GSEA enrichment analysis of data from the GSE196638 database and found that the glycolysis/gluconeogenesis pathway was upregulated in COPD patients compared to healthy controls (Figure 2A). Subsequently, Seahorse assays demonstrated an elevated extracellular acidification rate (ECAR) and a decreased oxygen consumption rate (OCR) in CSE‐treated primary mouse CD8^+^T cells (Figure 2B). The levels of glycolysis‐related enzymes ENO‐1, HK‐2, and LDHA were high in CSE‐treated primary mouse CD8^+^T cells (Figure 2C). Furthermore, the levels of lactate and pan‐lactylation (Pan‐Kla) were high in CSE‐treated primary mouse CD8^+^T cells (Figure 2D,E). These data indicate that CSE exposure induces the enhancement of glycolysis and the elevation of Pan‐Kla in CD8^+^T cells.

**FIGURE 2:**
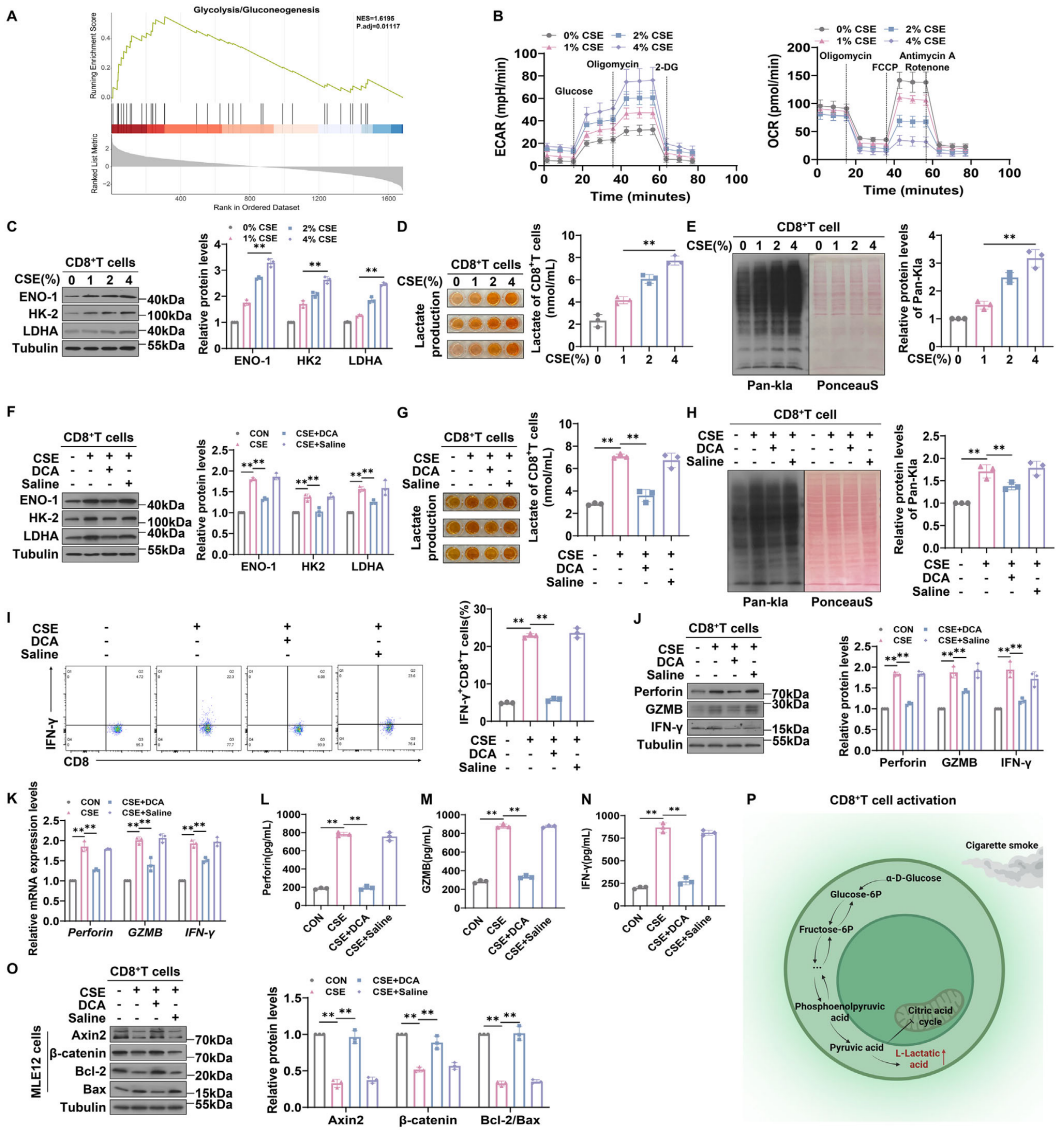
Glycolytic metabolic reprogramming is involved in CSE‐induced elevation of CD8^+^T cells activation and cytotoxicity. (A) GSEA enrichment analysis of glycolysis/gluconeogenesis pathways in CD8^+^T cells of healthy individuals and COPD patients. Primary CD8^+^T cells were exposed to 0%, 1%, 2%, or 4% CSE for 48 h. (B) Extracellular acidification rate (ECAR) and oxygen consumption rate (OCR) analysis of CD8^+^T cells. (C) Representative immunoblot images (left) and quantitative analysis (right) of ENO‐1, HK‐2, and LDHA in CD8^+^T cells. (D) Quantitative analysis of the levels of lactate in the medium of CD8^+^T cells. (E) Representative immunoblot images (left) and quantitative analysis (right) of pan‐lactate dehydrogenase (Pan‐Kla) in CD8^+^T cells. DCA (10 nM) or saline (0.9%) was added to CD8^+^T cells 6 h before 48‐h exposure to 0% or 4% CSE. (F) Representative immunoblot images (left) and quantitative analysis (right) of ENO‐1, HK‐2, and LDHA in CD8^+^T cells. (G) Quantitative analysis of the levels of lactate in the medium of CD8^+^T cells. (H) Representative immunoblot images (left) and quantitative analysis (right) of Pan‐Kla in CD8^+^T cells. (I) Representative flow cytometry profiles (left) and quantitative analysis (right) of IFN‐γ^+^CD8^+^T cells. (J) Representative immunoblot images (left) and quantitative analysis (right) of Perforin, GZMB, and IFN‐γ in CD8^+^T cells. (K) The levels of *Perforin*, *GZMB*, and *IFN‐γ* in CD8^+^T cells as measured by qRT‐PCR. Perforin (L), GZMB (M), and IFN‐γ (N) levels in the medium of CD8^+^T cells quantified by ELISA. (O) Representative immunoblot images (left) and quantitative analysis (right) of Axin2, β‐catenin, and Bcl‐2/Bax in MLE‐12 cells. (P) Schematic diagram illustrating the mechanism of glycolytic metabolic reprogramming in CD8^+^T cell activation. Data are presented as means ± SD, *n*  =  3 independent experiments. ^**^ *p* < 0.01, by one‐way ANOVA followed by Bonferroni correction.

Next, to determine if glycolytic metabolic reprogramming is involved in CSE‐induced activation of CD8^+^T cells, we used sodium dichloroacetate (DCA) to inhibit glycolysis. Immunoblot results demonstrated that DCA reversed the CSE‐induced elevations of ENO‐1, HK‐2, and LDHA levels (Figure 2F). DCA also blocked the increased levels of lactate and Pan‐Kla in CSE‐treated primary mouse CD8^+^T cells (Figure 2G,H). Further, we found via flow cytometry that DCA reversed the CSE‐induced elevation in IFN‐γ^+^CD8^+^T cells (Figure 2I). In primary mouse CD8^+^T cells, the CSE‐induced expression of Perforin, GZMB, and IFN‐γ was blocked by DCA (Figure 2J–N). Further, using the CD8^+^T cell‐MLE‐12 cell co‐culture model, we observed restoration of the Wnt/β‐catenin signaling‐related proteins Axin2 and β‐catenin, as well as apoptosis‐related proteins Bcl‐2 and Bax (Figure 2O). Consistently, intervention with the lactate dehydrogenase inhibitor GSK2837808A produced identical inhibitory effects on CSE‐triggered CD8^+^T cell metabolic reprogramming and hyperactivation, confirming that glycolysis‐derived lactate accumulation drives CD8^+^T cell‐mediated pulmonary epithelial injury (Figure S9). In contrast, downregulation of lactate receptor GPR81 did not block the CSE‐induced activation of CD8^+^T cells (Figure S10). In summary, these findings show that CSE induces glycolytic metabolic reprogramming in cells, leading to lactic acid accumulation and subsequent lactonization, which activates CD8^+^T cells (Figure 2P), and suggest an epigenetic role for lactylation in the activation of CD8^+^T cells.

### 2.3 Downregulation of AARS1 Ameliorates CSE‐Induced CD8^+^T Cell Activation

To elucidate the role of lactylation in CD8^+^T cells, we assessed the expression of lactylation‐related enzymes in these cells via scRNA‐seq analysis. The results demonstrated that AARS1, KAT7, and KAT8 were upregulated in CD8^+^T cells of COPD patients (Figure 3A; Figure S11). Next, we measured the levels of AARS1, KAT7, and KAT8 in CSE‐treated primary CD8^+^T cells and found that AARS1 was elevated, whereas KAT7 and KAT8 expression remained unchanged (Figure 3B,C). Moreover, silencing of AARS1 in primary CD8^+^T cells reversed the CSE‐induced elevation of Pan‐Kla levels (Figure 3D,E; Figure S12). Further, following AARS1 knockdown, the proportions of IFN‐γ^+^CD8^+^T cells were lower in CSE‐treated CD8^+^T cells (Figure 3F). AARS1 inhibition blocked the CSE‐induced upregulation of Perforin, GZMB, and IFN‐γ levels in CD8^+^T cells (Figure 3G,H). These findings were corroborated by cytokine ELISA of cell supernatants (Figure 3I–K). In the CD8^+^T cell and MLE‐12 cell co‐culture model, downregulation of AARS1 in CSE‐treated CD8^+^T cells restored MLE‐12 cell injury (Figure 3L). To exclude off‐target effects, a second independent AARS1‐targeting siRNA was applied, and highly consistent results were obtained across all functional assays (Figure S13). Therefore, these data show that the elevation of AARS1 contributes to CSE‐induced CD8^+^T cell activation.

**FIGURE 3:**
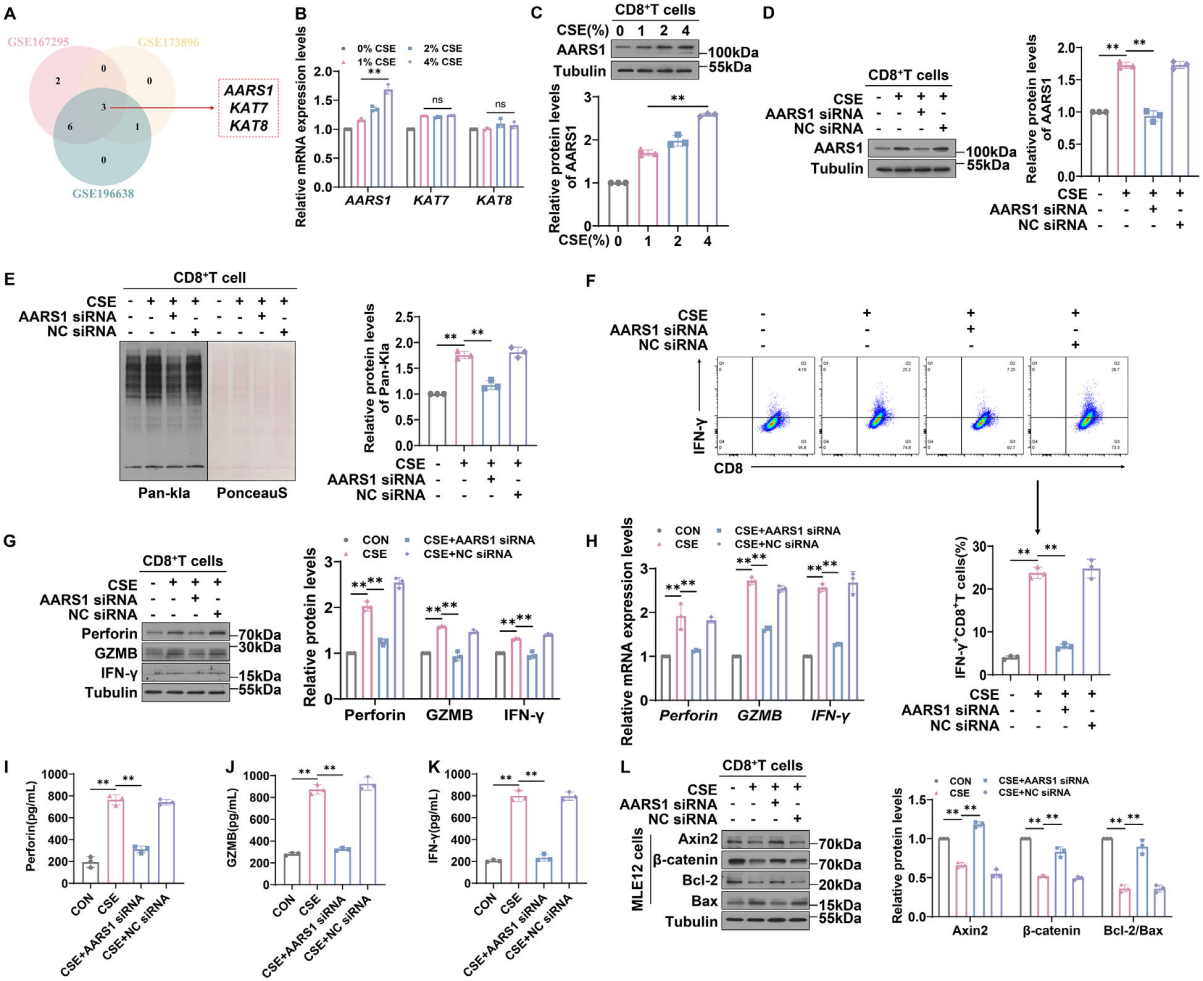
AARS1 is involved in CSE‐induced elevation of CD8^+^T cell activation and cytotoxicity. (A) Venn diagram showing the lactylation‐related enzymes in CD8^+^T cells from scRNA‐seq datasets GSE167295, GSE173896, and GSE196638. (B) The levels of *AARS1*, *KAT7*, and *KAT8* in CD8^+^T cells were measured by qRT‐PCR. (C) Representative immunoblot images (top) and quantitative analysis (bottom) of AARS1 in CD8^+^T cells. AARS1 siRNA (50 µM) or NC siRNA (50 µM) was added to CD8^+^T cells 6 h before 48‐h exposure to 0% or 4% CSE. (D) Representative immunoblot images (left) and quantitative analysis (right) of AARS1 in CD8^+^T cells. (E) Representative immunoblot images (left) and quantitative analysis (right) of Pan‐Kla in CD8^+^T cells. (F) Representative flow cytometry plots (top) and quantitative analysis (bottom) of IFN‐γ^+^CD8^+^T cells. (G) Representative immunoblot images (left) and quantitative analysis (right) of Perforin, GZMB, and IFN‐γ in CD8^+^T cells. (H) The levels of *Perforin*, *GZMB*, and *IFN‐γ* in CD8^+^T cells were measured by qRT‐PCR. Perforin (I), GZMB (J), and IFN‐γ (K) levels in the medium of CD8^+^T cells were quantified by ELISA. (L) Representative immunoblot images (left) and quantitative analysis (right) of Axin2, β‐catenin, and Bcl‐2/Bax in MLE‐12 cells. Data are presented as means ± SD, *n*  =  3 independent experiments. ^**^ *p* < 0.01, by one‐way ANOVA followed by Bonferroni correction. ns, not significant.

### 2.4 RUNX3 Lys193 Lactylation Inhibits Its Autophagolysosomal Degradation in CSE‐Treated CD8^+^T Cells

To elucidate which protein AARS1 regulates to activate CD8^+^T cells, we performed proteomics to identify significantly changed proteins in CSE‐treated CD8^+^T cells transfected with AARS1 siRNA. Overlaps between the AARS1‐influenced proteins in proteomics, lactylation proteomics, and proteins associated with CD8^+^T cell activation are shown in a Venn diagram. Prominent proteins were Lat, RUNX3, and PCIF1(Figure 4A). Next, we demonstrated that, in CSE‐treated primary CD8^+^T cells, RUNX3 expression was upregulated, but not the expression of Lat or PCIF1 (Figure 4B). Downregulation of AARS1 reduced the high levels of RUNX3 in CSE‐treated primary CD8^+^T cells (Figure 4C). Reciprocal IP experiments confirmed the physical interaction between AARS1 and RUNX3, and CSE exposure enhanced their binding interaction in CD8^+^T cells (Figure 4D). Based on IP assays, we identified the elevation of lactylation on RUNX3 in CSE‐treated primary CD8^+^T cells (Figure 4E; Figure S14). To further clarify the modification specificity, we additionally examined the acetylation and methylation levels of RUNX3 upon CSE treatment (Figure S15A,B). We found that CSE treatment specifically enhanced RUNX3 lactylation, while no obvious alterations were observed in RUNX3 acetylation or methylation. Further, overexpression of AARS1 induced the elevation of RUNX3 lactylation in CD8^+^T cells (Figure S15C,D). Protein degradation in cells primarily occurs via two pathways, the ubiquitin‐proteasome system (UPS) and the autophagy‐lysosomal pathway [22]. Therefore, we designed experiments to delineate the specific degradation pathway responsible for RUNX3 regulation in CD8^+^T cells. First, we assessed RUNX3 half‐life via a cycloheximide (CHX) assay and found slowed degradation in CSE‐treated cells compared to controls (Figure 4F). Further, exposure of CD8^+^T cells to the proteasome inhibitor MG132 revealed no appreciable changes in levels of the RUNX3 protein (Figure 4G). RUNX3 levels increased upon exposure of CD8^+^T cells to the autophagy inhibitor chloroquine (CQ) (Figure 4H). IF assays validated this finding in CSE‐treated CD8^+^T cells. Autophagy marker levels (LC3B) showed no obvious alterations, whereas RUNX3 expression was upregulated (Figure 4I). These results indicate that CSE suppresses RUNX3 degradation in CD8^+^T cells via inhibiting its autolysosomal fusion.

**FIGURE 4:**
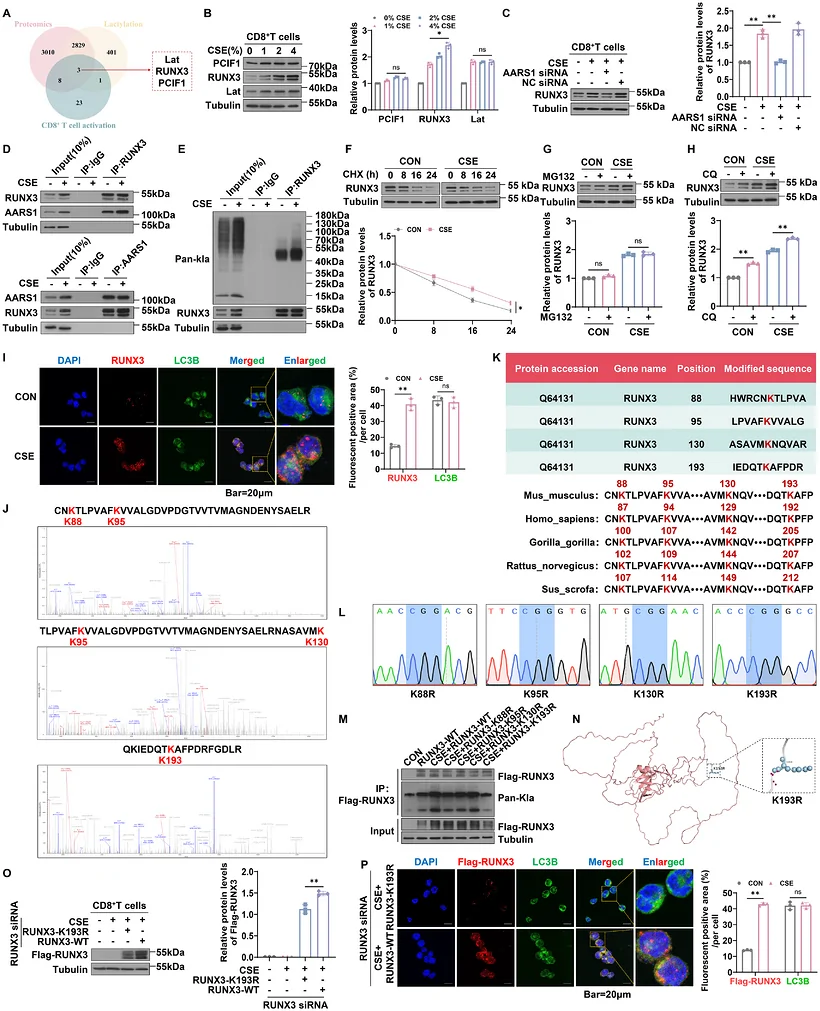
AARS1 mediates RUNX3‐K193 lactylation and its autophagic lysosomal degradation in CSE‐treated CD8^+^T cells. (A) Venn diagram showing datasets for proteomics, lactylation sequencing, and proteins associated with CD8^+^T cell activation. (B) Representative immunoblot images (left) and quantitative analyses (right) of PCIF1, RUNX3, and Lat in CD8^+^T cells. AARS1 siRNA (50 µM) or NC siRNA (50 µM) was added to CD8^+^T cells 6 h before 0% or 4% CSE exposure for 48 h. (C) Representative immunoblot images (top) and quantitative analysis (bottom) of RUNX3 in CD8^+^T cells. (D) Reciprocal immunoprecipitation (IP) assay to validate the interaction between AARS1 and RUNX3 in CD8^+^T cells. (E) IP validation of RUNX3 lactylation in CD8^+^T cells with 0% or 4% CSE exposure. (F) CHX (20 µg/mL) was added to CD8^+^T cells exposed to 0% or 4% CSE for 0, 8, 16, or 24 h. Representative immunoblot images (top) and quantification (bottom) of RUNX3 in CD8^+^T cells. MG132 (10 µm) or CQ (50 µm) was added to CD8^+^T cells 24 h before 0% or 4% CSE exposure. After MG132 exposure (G) or CQ exposure (H), representative immunoblot images (top) and quantification (bottom) of RUNX3 in CD8^+^T cells. (I) Representative immunostaining images (left) and quantitative analyses (right) of RUNX3 and LC3B in CD8^+^T cells. Red‐RUNX3, green‐LC3B, blue‐DAPI. Scale bar = 20 µm, magnification = 1000x. (J) LC‐MS/MS validation of RUNX3 lactylation and identification of its modification sites (K88, K95, K130, K193). (K) Homology analysis of RUNX3 potential lactylation sites across various species (mouse, *homo sapiens*, chimpanzee, rat, wild boar). (L) Overlap‐PCR for constructing mutant plasmids. Sanger sequencing validating the lysine‐to‐arginine mutation (CGG). (M) IP showing mutant plasmid re‐expression, and immunoblotting assessed Flag‐RUNX3 lactylation in CD8^+^T cells. (N) Prediction of RUNX3 3D structure with AlphaFold and visualization of the K193R mutation site via PyMOL. RUNX3 was silenced in CD8^+^T cells, followed by transfection with RUNX3‐WT or RUNX3‐K193R plasmids before 4% CSE exposure. (O) Representative immunoblot images (left) and quantitative analysis (right) of Flag‐RUNX3 in CD8^+^T cells. (P) Representative immunostaining images (left) and quantitative analyses (right) of Flag‐RUNX3 and LC3B in CD8^+^T cells. Red‐Flag‐RUNX3, green‐LC3B, blue‐DAPI. Scale bar = 20 µm, magnification = 1000x. Data are presented as means ± SD, *n*  =  3 independent experiments. ^*^ *p* < 0.05, ^**^ *p* < 0.01, by one‐way ANOVA followed by Bonferroni correction. ns, not significant.

To clarify the role of lactylation on RUNX3, specific lactylation modification sites in the RUNX3 protein were identified using LC‐MS/MS. The results revealed that K88, K95, K130, and K193 were potential lactylation sites (Figure 4J). Sequence alignment across species showed that these four sites were conserved (Figure 4K). Through site‐directed mutations, we found that K193 serves as the primary lactylation site on RUNX3 (Figure 4L,M). The 3D structure of RUNX3 was predicted using the AlphaFold website. The K193R mutation site in RUNX3 was visualized by PyMOL (Figure 4N). We then knocked down RUNX3 in CD8^+^T cells, transfected RUNX3‐K193R or RUNX3‐WT, and exposed the cells to CSE. RUNX3‐K193 knockdown reversed CSE‐induced upregulation of Flag‐RUNX3 (Figure 4O; Figure S16A,B). Treating CD8^+^T cells in the same manner revealed that the Flag‐positive area exhibited identical changes, restoring its function of autolysosomal degradation (Figure 4P). Thus, these data identify K193 as the primary lactylation site of RUNX3 and show that this site mediates its autolysosomal function.

### 2.5 AARS1 Mediates RUNX3 Lys193 Lactylation, Which Is Involved in CSE‐Induced CD8^+^T Cell Activation

Our previous findings demonstrated that elevated AARS1 induced lactylation of RUNX3 at Lys 193, thereby inhibiting its autolysosomal degradation. Therefore, to elucidate the impact of lactyl modification at the K193 site on CD8^+^T cell activation, we conducted the following studies. For the RUNX3 site‐mutation assay, endogenous RUNX3 was knocked down in CD8^+^T cells, followed by transfection with RUNX3‐WT or RUNX3‐K193R plasmids. Cells were then stimulated with 0% or 4% CSE for 48 h. We observed decreased RUNX3 lactylation in the RUNX3‐K193R group. Further, the total RUNX3 protein abundance was higher in the RUNX3‐WT input group, demonstrating that K193 lactylation enhanced the stability of RUNX3 protein in CSE‐treated CD8^+^T cells (Figure 5A). Flow cytometry analyses provided additional evidence that the proportions of IFN‐γ^+^CD8^+^ T cells were lower in the RUNX3‐K193 mutant group versus the CSE‐treated group (Figure 5B). Following mutation of RUNX3‐K193, mRNA and protein expression levels of Perforin, GZMB, and IFN‐γ were restored in CSE‐treated CD8^+^T cells (Figure 5C,D). ELISA tests confirmed these findings (Figure 5E–G). To further define the underlying transcriptional mechanism, we performed ChIP‐qPCR assays to evaluate the binding of RUNX3 to the promoters of these cytotoxic effector genes. The results demonstrated that CSE facilitates the transcriptional activation of Perforin, GZMB, and IFN‐γ by enhancing the promoter occupancy of RUNX3 (Figure S17). Co‐culture of CD8^+^T cells expressing mutant RUNX3‐K193 with MLE‐12 cells restored the regenerative and anti‐apoptotic capacity of MLE‐12 cells post‐mutation and reduced apoptosis (Figure 5H). We performed the same series of experiments after knocking down RUNX3, yielding consistent results (Figure S16C–J). These findings indicate that lactylation of RUNX3 at Lys 193 promotes CD8^+^T cell activation and enhances their cytotoxicity.

**FIGURE 5:**
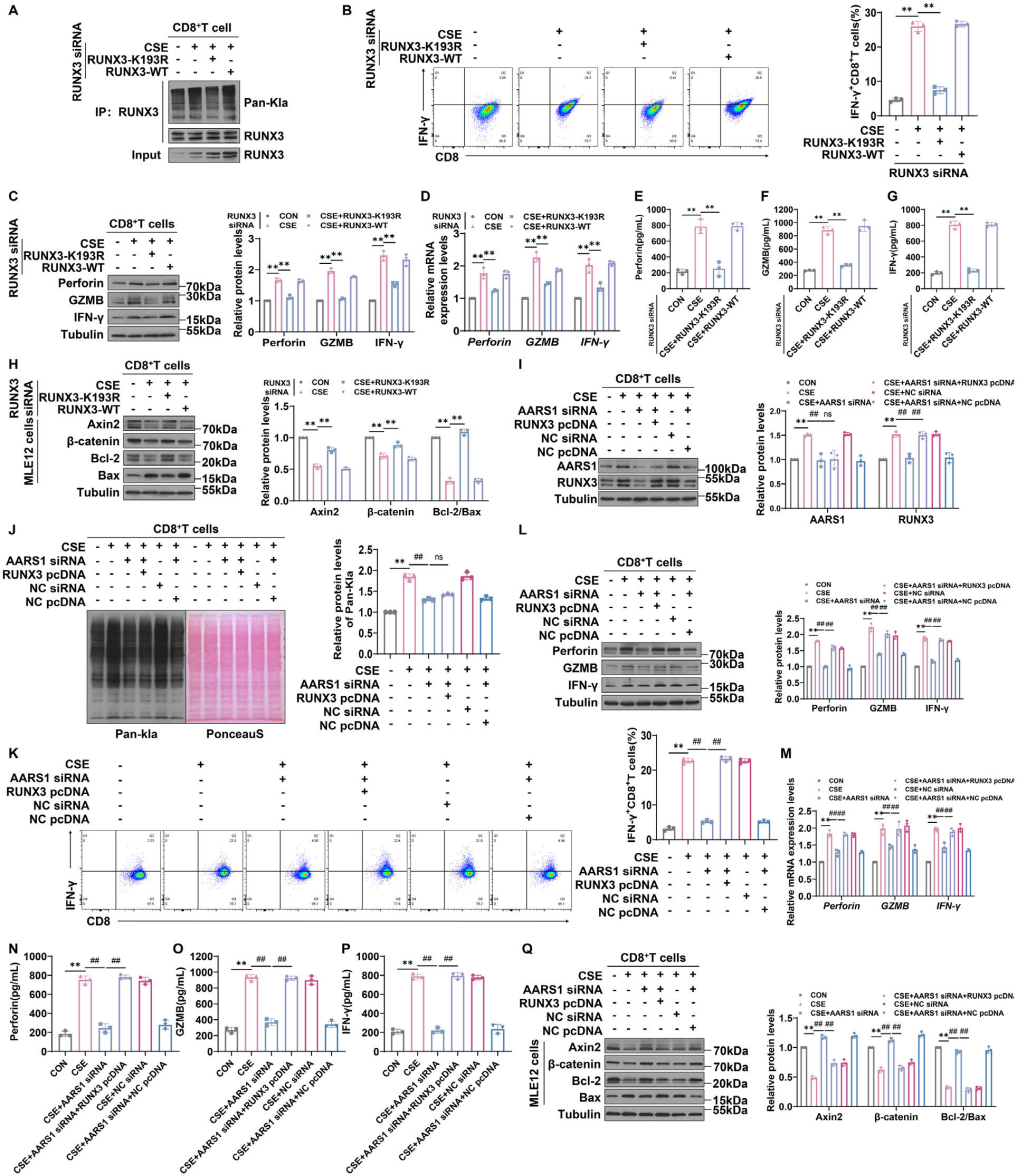
AARS1 mediation of RUNX3‐K193 lactylation contributes to CSE‐induced elevation of CD8^+^T cell activation and cytotoxicity. RUNX3 was silenced in CD8^+^T cells, followed by transfection with RUNX3‐WT or RUNX3‐K193R plasmids before 48 h of exposure to 0% or 4% CSE. (A) IP validation of RUNX3 lactylation in CD8^+^T cells. (B) Representative flow cytometry profiles (left) and quantitative analysis (right) of IFN‐γ^+^CD8^+^ T cells. (C) Representative immunoblot images (left) and quantitative analysis (right) of Perforin, GZMB, and IFN‐γ in CD8^+^T cells. (D) The levels of *Perforin*, *GZMB*, and *IFN‐γ* in CD8^+^T cells were measured by qRT‐PCR. Perforin (E), GZMB (F), and IFN‐γ (G) levels in the medium of CD8^+^T cells were quantified by ELISA. (H) Representative immunoblot images (left) and quantitative analysis (right) of Axin2, β‐catenin, and Bcl‐2/Bax in MLE‐12 cells. Following 6 h of transfection with AARS1 siRNA or RUNX3 pcDNA, CD8^+^T cells underwent 48 h of 0% or 4% CSE exposure. (I) Representative immunoblot images (left) and quantitative analysis (right) of AARS1 and RUNX3 in CD8^+^T cells. (J) Representative immunoblot images (left) and quantitative analysis (right) of Pan‐Kla in CD8^+^T cells. (K) Representative flow cytometry profiles (left) and quantitative analysis (right) of IFN‐γ^+^CD8^+^T cells. (L) Representative immunoblot images (left) and quantitative analysis (right) of Perforin, GZMB, and IFN‐γ in CD8^+^T cells. (M) The levels of *Perforin*, *GZMB*, and *IFN‐γ* in CD8^+^T cells measured by qRT‐PCR. Perforin (N), GZMB (O), and IFN‐γ (P) levels in the medium of CD8^+^T cells quantified by ELISA. (Q) Representative immunoblot images (left) and quantitative analysis (right) of Axin2, β‐catenin, and Bcl‐2/Bax in MLE‐12 cells. Data are presented as means ± SD, *n*  =  3 independent experiments. ^**^ *p* < 0.01, ^##^ *p* < 0.01, by one‐way ANOVA followed by Bonferroni correction. n.s., not significant.

To explore the regulatory role of AARS1‐mediated RUNX3‐K193 lactylation in CD8^+^T cell activation, we transfected AARS1 siRNA and RUNX3 pcDNA into CSE‐treated CD8^+^T cells, with RUNX3 pcDNA being utilized to induce RUNX3 overexpression in the target cells (Figure S18). In CSE‐treated CD8^+^T cells, AARS1 siRNA knockdown prevented the increase in RUNX3. However, RUNX3 overexpression reversed this effect (Figure 5I). CSE‐induced Pan‐Kla levels were reduced by AARS1 knockdown, but this effect was reversed upon RUNX3 overexpression (Figure 5J). Flow cytometry analysis revealed that the CSE‐induced increase in the proportion of IFN‐γ^+^CD8^+^T cells was reduced upon AARS1 knockdown, but the proportion of CSE‐induced IFN‐γ^+^CD8^+^T cells increased after RUNX3 overexpression (Figure 5K). Furthermore, AARS1 knockdown in CSE‐treated CD8^+^T cells reduced Perforin, GZMB, and IFN‐γ expression at both protein and mRNA levels (Figure 5L,M), an effect reversed by RUNX3 upregulation. ELISA confirmed that co‐transfection of AARS1 siRNA and RUNX3 pcDNA restored the diminished levels of these factors in cell supernatants, relative to AARS1 knockdown alone (Figure 5N–P). AARS1 knockdown restored the expression of Wnt/β‐catenin signaling‐related proteins (Axin2 and β‐catenin) and apoptosis‐related proteins (Bcl‐2 and Bax). In contrast, RUNX3 overexpression diminished these protective effects and promoted MLE‐12 cell injury (Figure 5Q). These data demonstrate that, in CSE‐treated CD8^+^T cells, AARS1 mediates the activation of CD8^+^T cells and the enhancement of cytotoxicity via RUNX3.

### 2.6 DCA or the AARS1 Inhibitor Suppresses CSE‐Induced CD8^+^T Cell Activation and Alleviates Injury to Lung Organoids in the CD8^+^ T Cell‐Lung Organoid Co‐Culture System

In both basic and clinical research, organoids are useful tools, offering substantial utility in disease modeling, drug discovery, and therapeutic screening due to their capacity to recapitulate tissue architecture and physiological functions [23]. Therefore, to investigate the functional impact of CSE‐treated CD8^+^T cells on alveolar homeostasis, we established a co‐culture model of primary CD8^+^T cells and mouse lung organoids following our previously published protocols [24] (Figure 6A). Prior to co‐culture experiments, we validated the morphological integrity of the lung organoids via light microscopy and confirmed the presence of surfactant protein C^+^ (SP‐C^+^) alveolar epithelial cells through IF staining, ensuring that the organoids retained key alveolar phenotypic features (Figure 6B,C). Co‐culture with CSE‐treated CD8^+^T cells inhibited lung organoid growth; this growth‐suppressive effect was reversed by either DCA (a glycolysis inhibitor) or AARS1 siRNA intervention (Figure 6D,F). The percentage of SP‐C^+^ cells decreased in the mouse lung organoids co‐cultured with CSE‐treated CD8^+^T cells, and this decline was minimized by DCA administration or AARS1 siRNA intervention (Figure 6E,G). We examined the expression of Wnt/β‐catenin signaling‐related proteins (Axin2 and β‐catenin) and apoptosis‐related proteins (Bcl‐2 and Bax) in lung organoids, revealing that DCA or AARS1 silencing blocked CSE‐induced upregulation of Bax and restored the downregulation of Axin2, β‐catenin, and Bcl‐2 (Figure 6H,I). These findings demonstrate that targeting lactate metabolism via DCA or AARS1‐mediated lactylation alleviates CSE‐induced alveolar epithelial injury and impaired regeneration, suggesting a therapeutic strategy for smoking‐related emphysema.

**FIGURE 6:**
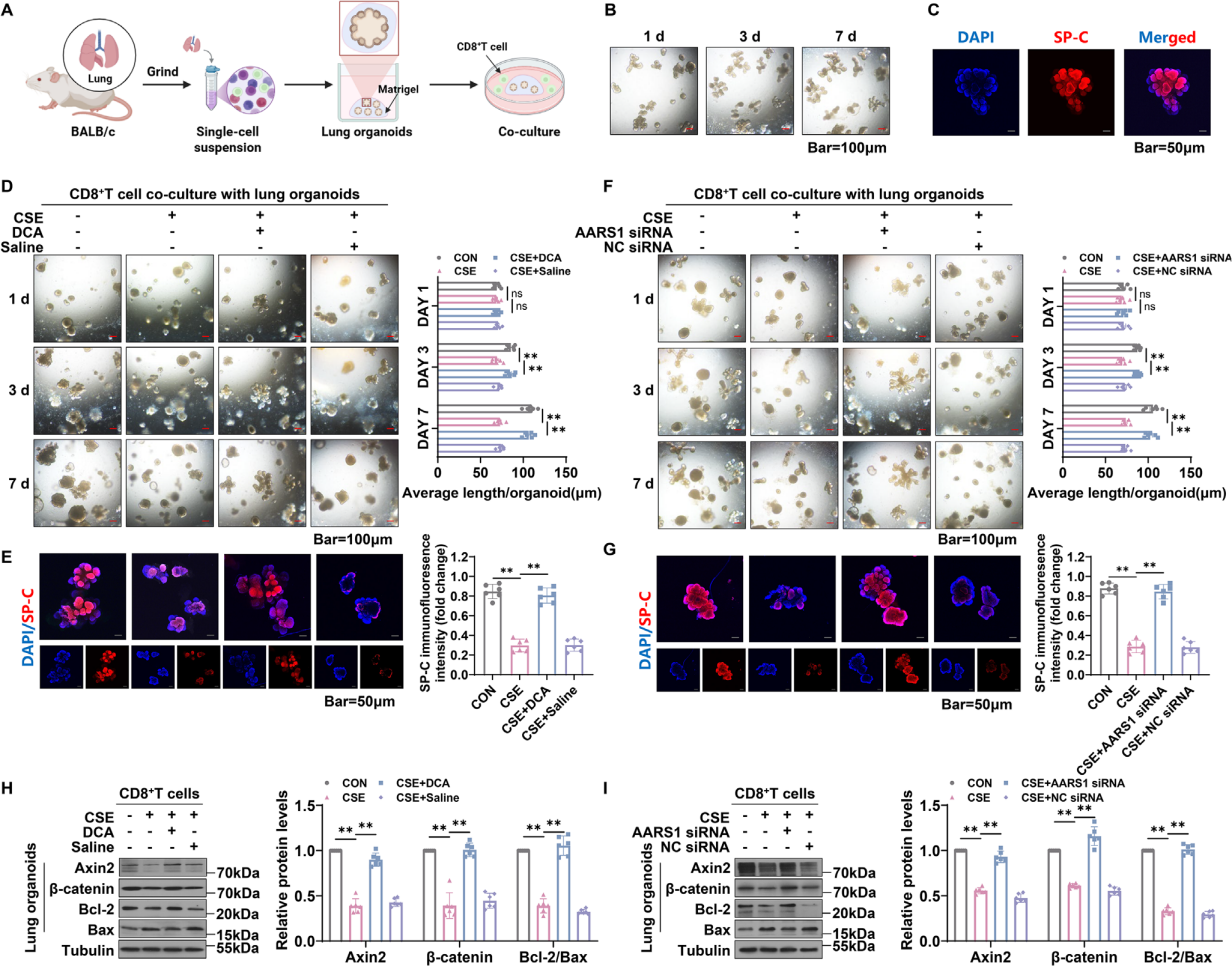
CSE‐induced elevation of CD8^+^T cell activation and cytotoxicity is inhibited by DCA or by an AARS1 inhibitor in CD8^+^T cells co‐cultured with mouse lung organoids. (A) Schematic diagram of the CD8^+^T cell‐co‐cultured mouse lung organoid model. (B) Representative light microscopy (left) (scale bar, 100 µm) of mouse lung organoids at days 1, 3, and 7. (C) Representative SP‐C immunostaining images (right) (scale bar, 50 µm) of mouse lung organoids. At 12 h after CD8^+^T cells were exposed to DCA (10 nM) or AARS1 siRNA (50 µM), they were exposed to 0% or 4% CSE for 48 h and were co‐cultured with mouse lung organoids for 7 days. (D) Representative light microscopy (left, scale bar, 100 µm) and mean diameters (right) of mouse lung organoids at days 1, 3, and 7. (E) SP‐C immunostaining (scale bar, 50 µm) images of mouse lung organoids at day 7. (F) Representative light microscopy (left, scale bar, 100 µm) and mean diameters (right) of mouse lung organoids at days 1, 3, and 7. (G) SP‐C immunostaining images (left, scale bar, 50 µm) and quantification (right) of mouse lung organoids at day 7. (H) Representative immunoblot images (left) and quantitative analyses (right) of Axin2, β‐catenin, and Bcl‐2/Bax in mouse lung organoids (DCA treatment). (I) Representative immunoblot images (left) and quantitative analyses (right) of Axin2, β‐catenin, and Bcl‐2/Bax in mouse lung organoids (AARS1 knockdown). Data are presented as means ± SD, *n*  =  6 independent experiments. ^**^ *p* < 0.01, by one‐way ANOVA followed by Bonferroni correction. n.s., not significant.

### 2.7 DCA Alleviates CD8^+^T Cell Activation in Mice with CS‐Induced Emphysema

To investigate the effects of lactic acid on CS‐induced emphysema in mice, we inhibited lactic acid production in mouse lung tissue via intraperitoneal injection of DCA. Six‐week‐old BALB/c male mice were exposed to CS while receiving DCA for 24 weeks (Figure 7A). First, we quantified lactate levels in mouse serum, finding that DCA treatment reversed the CS‐induced elevation in serum lactate compared to the CS‐exposed group (Figure 7B; Figure S19). DCA treatment also minimized the global upregulation of Pan‐Kla in mouse lungs (Figure 7C). Further histological and cellular analyses showed that DCA alleviated CS‐induced alveolar wall destruction, as evidenced by a reduction in mean linear intercept (MLI), and inhibited CS‐triggered alveolar epithelial cell apoptosis, as reflected by increased SP‐C^+^ alveolar epithelial cell counts in lungs (Figure 7D,E). At the molecular level, DCA prevented CS‐induced Bax upregulation and restored the expressions of Axin2, β‐catenin, and Bcl‐2 (Figure 7F). Compared to the CS group, DCA diminished the infiltration of CD8a^+^ cells and the proportion of IFN‐γ^+^CD8^+^T cells in the lungs of mice (Figure 7G,H). There were lower serum levels of CD8^+^T cell‐derived cytotoxic factors, including Perforin, GZMB, and IFN‐γ (Figure 7I–K). Thus, these data demonstrate that targeted regulation of lactate metabolism in mice diminishes CD8^+^T cell activation and cytotoxicity, thus providing protection against CS‐elicited emphysema.

**FIGURE 7:**
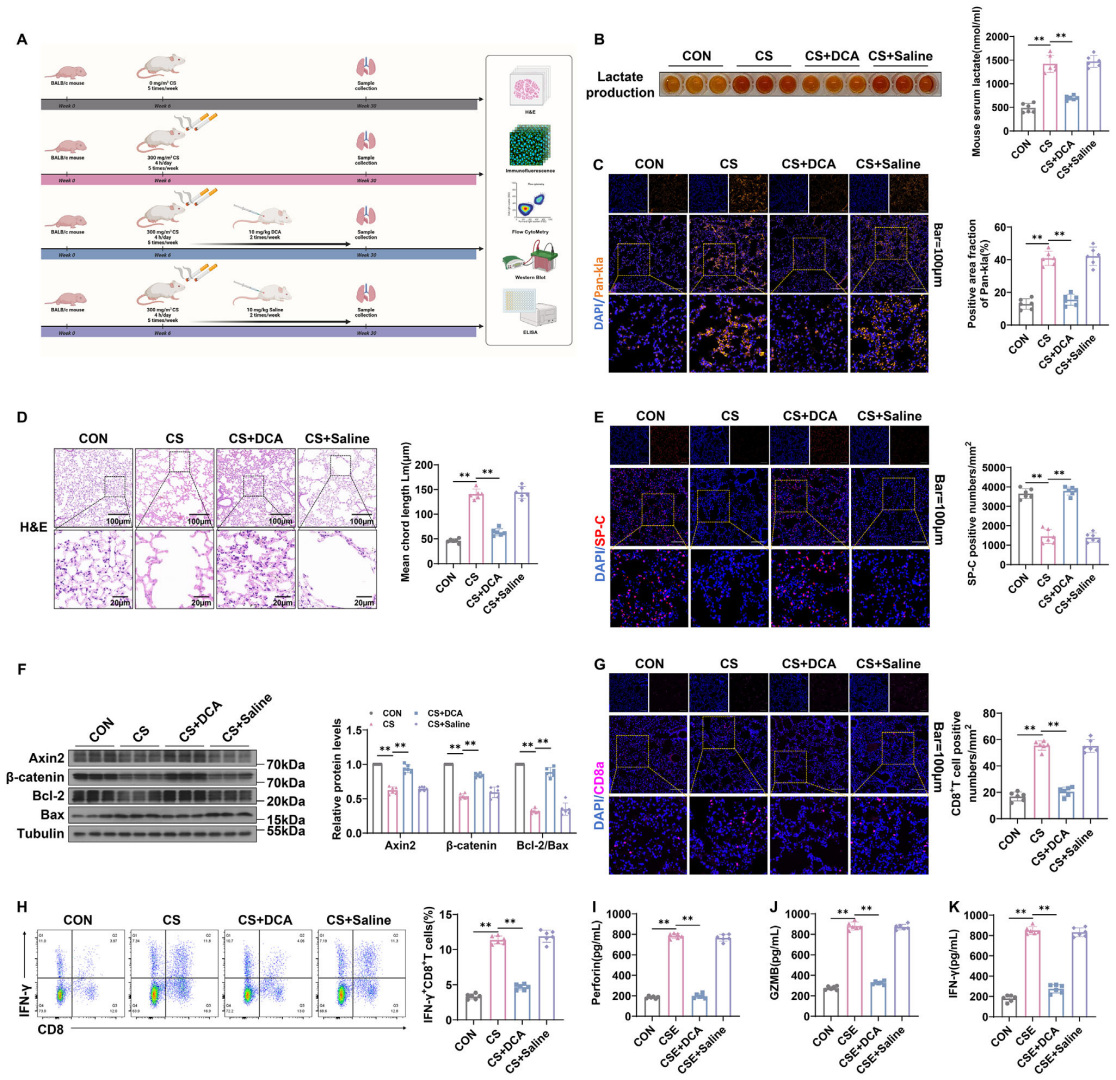
DCA reduces emphysema through inhibition of CD8^+^T cell activation in CS‐exposed mice. Male BALB/c mice were exposed to air or CS (300 mg/m^3^ TPM) and administered either 10 mg/kg DCA or not treated. (A) Schematic diagram of mouse model. (B) Representative images (left) and quantification (right) of lactate in lungs of mice. (C) Representative immunostaining images (left) and quantitative analysis (right) of Pan‐Kla in lungs from mice (scale bar, 100 µm). (D) Representative H&E‐stained (left) and quantitative analysis (right) in lungs of mice (scale bars: 100 µm and 20 µm). (E) Representative immunostaining images (left) and quantitative analysis (right) of SP‐C in lungs of mice (scale bar, 100 µm). (F) Representative immunoblot images (left) and quantitative analysis (right) of Axin2, β‐catenin, and Bcl‐2/Bax in mouse lungs. (G) Representative immunostaining images (left) and quantitative analysis (right) of CD8a in mouse lungs (scale bar, 100 µm). (H) Representative flow cytometry plots (left) and quantitative analysis (right) of IFN‐γ^+^CD8^+^T cells isolated from mouse lungs. Perforin (I), GZMB (J), and IFN‐γ (K) levels in serum of mice quantified by ELISA. Data are presented as means ± SEM, *n*  =  6 biological replicates. ^**^ *p* < 0.01, by one‐way ANOVA followed by Bonferroni correction.

### 2.8 _β_LNPs@AARS1‐siRNA Attenuates CD8^+^T Cell Activation in Mice with CS‐Induced Emphysema

Lipid nanoparticles (LNPs) are small particles (ranging from 1 to 300 nm) that can carry drugs, oligonucleotides, peptides, or other cargo and deliver them to target tissues. Various LNPs have been employed for siRNA delivery in biomedical applications [25]. Furthermore, lipid particles coated with β‐sitosterol and polyethylene glycol (PEG) to deliver specific siRNAs provide gene silencing at lower siRNA concentrations than required by the original LNP formulation, thereby reducing toxicity [26]. Therefore, we selected LNPs to carry AARS1 siRNA. Six‐week‐old BALB/c mice were exposed to CS while receiving intranasal instillation of _β_LNPs@AARS1‐siRNA for 24 weeks to target CD8^+^T cells in mouse lungs and achieve AARS1 knockdown (Figure 8A,B). Transmission electron microscopy and Malvern particle size analysis revealed that the prepared LNPs had sizes of 160 to 170 nm (Figure 8C–E). The LNPs exhibited a zeta potential of 30 mV to 40 mV and a polydispersity index (PDI) of 0.15 to 0.2 (Figure 8F,G), indicating sufficient suspension and dispersion properties. IF analysis of mouse lungs following intranasal LNP instillation validated the LNP delivery efficiency (Figure 8H). Additionally, to validate the reduction of AARS1 in CD8^+^T cells targeted by _β_LNPs@AARS1‐siRNA, IF colocalization revealed a decrease in AARS1 and CD8a colocalization‐positive cells following _β_LNPs@AARS1‐siRNA treatment (Figure 8I). H&E, IF, and WB analyses demonstrated that _β_LNPs@AARS1‐siRNA mitigated CS‐induced damage to mouse alveolar walls, as evidenced by increased mean linear cross‐sectional diameters (Figure 8J) and alveolar epithelial cell apoptosis, primarily manifested by increased SP‐C^+^ cell numbers (Figure 8K). It also blocked elevated Bax protein levels and restored CS‐induced downregulation of Axin2, β‐catenin, and Bcl‐2 (Figure 8L). Similarly, _β_LNPs@AARS1‐siRNA reduced CD8a^+^ cell numbers and decreased the proportions of IFN‐γ^+^CD8^+^T cells in mouse lungs compared to the CS‐exposed group (Figure 8M,N). There were lower levels of cytotoxic factors, including Perforin, GZMB, and IFN‐γ in mouse serum (Figure 8O–Q). These data demonstrate that targeting of AARS1 in mice suppresses CD8^+^T cell activation and cytotoxicity, thereby protecting against CS‐induced emphysema.

**FIGURE 8:**
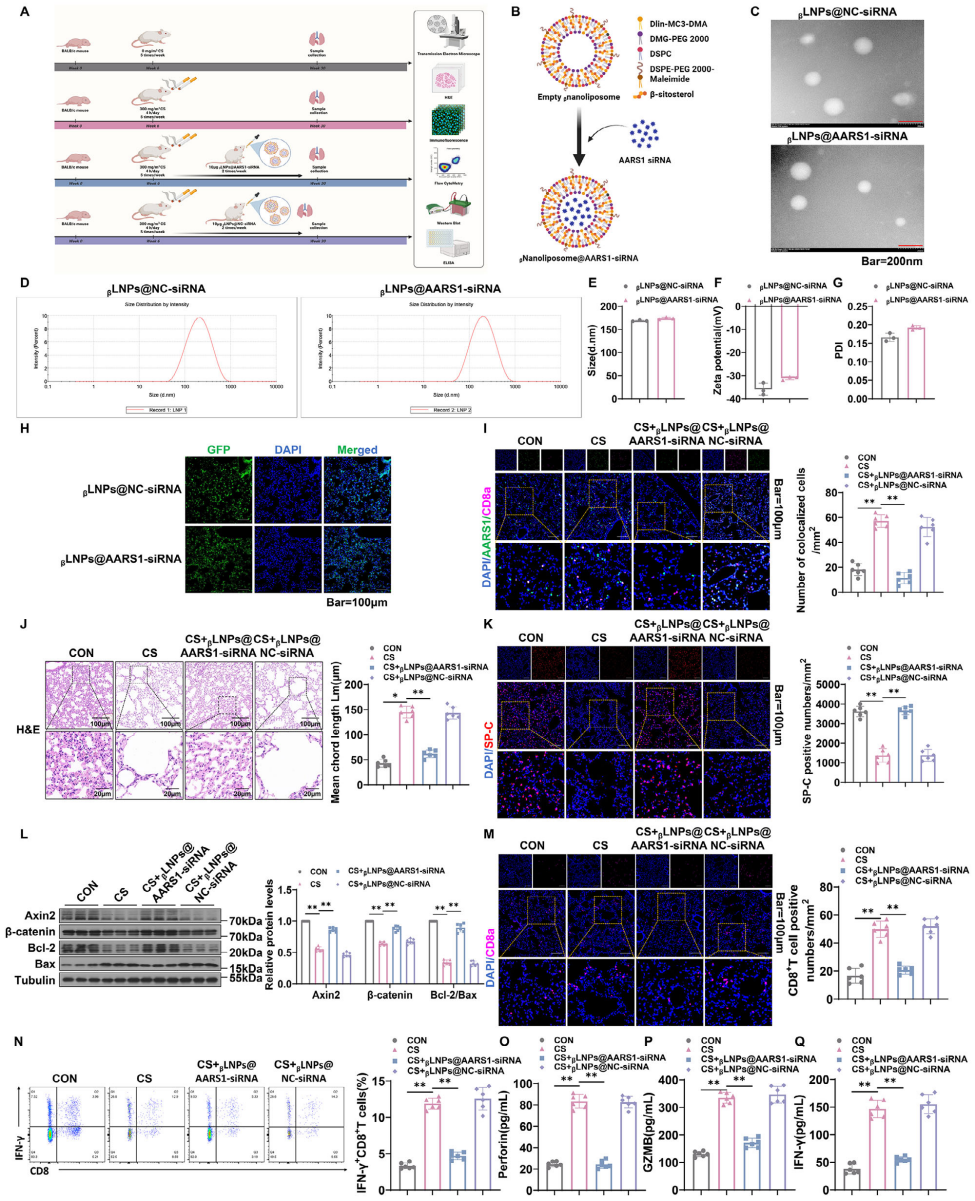
_β_LNPs@AARS1‐siRNA reduces emphysema through inhibition of CD8^+^T cell activation in CS‐exposed mice. Male BALB/c mice were exposed to air or CS (300 mg/m^3^ TPM) and administered either 10 µg _β_lipid nanoparticles (_β_LNPs) @AARS1‐siRNA or not treated. AARS1 siRNA was encapsulated into _β_LNPs to form AARS1 siRNA‐loaded _β_LNPs, which were used for delivery of AARS1 siRNA to mice to achieve AARS1 knockdown. (A) Schematic diagram of mouse model. (B) Schematic of drug‐loaded _β_LNP construction. (C) Transmission electron microscopy (TEM) image of _β_LNPs showing particle sizes (scale bar: 200 nm). (D) Schematic of particle size distribution of _β_LNPs@AARS1‐siRNA and _β_LNPs@NC‐siRNA measured by a Malvern particle size analyzer. Particle size distribution (E), zeta potential (F), and polydispersity index (G) of _β_LNPs@AARS1‐siRNA and _β_LNPs@NC‐siRNA. (H) Immunostaining images showing the infection efficiency of _β_LNPs in lungs from mice (scale bar, 100 µm). (I) Representative immunostaining images (left) and quantitative analysis (right) of AARS1/CD8a co‐localization in lungs of mice (scale bar, 100 µm). (J) Representative H&E‐stained (left) and quantitative analysis (right) of lung sections of mice (scale bars: 100 µm and 20 µm). (K) Representative immunostaining images (left) and quantitative analysis (right) of SP‐C in mouse lungs (scale bar, 100 µm). (L) Representative immunoblot images (left) and quantitative analyses (right) of Axin2, β‐catenin, and Bcl‐2/Bax in mouse lungs. (M) Representative immunostaining images (left) and quantitative analysis (right) of CD8a in mouse lungs (scale bar, 100 µm). (N) Representative flow cytometry plots (left) and quantitative analysis (right) of IFN‐γ^+^CD8^+^T cells isolated from mouse lungs. Perforin (O), GZMB (P), and IFN‐γ (Q) levels in serum of mice were quantified by ELISA. Data are presented as means ± SEM, *n*  =  6 biological replicates. ^*^ *p* < 0.05, ^**^ *p* < 0.01, by one‐way ANOVA followed by Bonferroni correction.

### 2.9 The Association Between CD8^+^T Cell Activation and COPD Progression

Peripheral blood serum samples were collected from age‐ and sex‐matched healthy controls and clinically diagnosed COPD patients (Table 1) to quantify levels of lactate, AARS1, and CD8^+^T cell‐derived cytotoxic cytokines. Our findings demonstrated that in patients with COPD compared with healthy controls, the number of Pan‐Kla/CD8a double‐positive cells identified by immunofluorescence colocalization was increased, and serum lactate concentrations were elevated (Figure 9A,B). Linear regression analysis revealed negative correlations between serum lactate levels and two key lung function metrics: forced expiratory volume in 1 s/forced vital capacity ratio (FEV1/FVC, %) and percentage of predicted FEV1 (FEV1%) (Figure S20). We validated the dysregulated CD8^+^T cell phenotype in lung tissues. Consistent with our previously observed upregulation of Pan‐Kla and AARS1 in pulmonary CD8^+^T cells, RUNX3 expression was elevated in lung‐resident CD8^+^T cells from COPD patients and exhibited a negative correlation with FEV1/FVC (%) and predicted FEV1 (%) (Figure S21). Consistently, immunofluorescence colocalization showed that the number of AARS1/CD8a double‐positive cells was increased (Figure 9C), and ELISA demonstrated elevated serum AARS1 levels in COPD patients (Figure 9D). Both showed a negative correlation with the above two lung function parameters in our study cohort (Figure 9E). Compared with healthy controls, serum levels of the cytotoxic effector molecules Perforin, GZMB, and IFN‐γ were significantly increased in COPD patients. Further correlation analysis showed that, for COPD patients, serum AARS1 levels positively correlated with the cytotoxic effector molecules Perforin, GZMB, and IFN‐γ, verifying that, during COPD progression, AARS1 upregulation is coupled with the enhanced cytotoxic activity of CD8^+^T cells (Figure S22). Moreover, compared with healthy controls, serum concentrations of the cytotoxic effector molecules Perforin, GZMB, and IFN‐γ were upregulated in COPD patients (Figure 9F,H,J). Further regression analysis confirmed that the peripheral levels of these molecules showed negative correlations with FEV1/FVC (%) and predicted FEV1 (%) (Figure 9G,I,K). These clinical data demonstrate that, for COPD patients, CD8^+^T cells are functionally activated, highlighting their potential pathogenic role in driving disease progression.

**TABLE 1:** Clinical data for the serum donors.

	Healthy individuals	COPD patients
Number	21	15
Sex(male)	20 (95.23%)	13 (86.67%)
Age(years)	63.81 ± 13.19	68.47 ± 6.79^a^
BMI	24.60 ± 2.72	20.03 ± 2.58^a^
Smoking history (pack‐years)	—	60.60 ± 44.97
GOLD grade, I/II/III/IV	—	9/3/2/1
FEV_1_ (%) predicted	87.69 ± 11.75	38.91 ± 14.90^b^
FEV_1_/FVC (%)	89.13 ± 8.39	46.51 ± 13.45^b^

^a^ *p* < 0.05 versus healthy group.

^b^ *p* < 0.01 versus healthy group.

Data are presented as means ± SD, unless otherwise stated.

Descriptive data for the intervention and comparison groups were compared using the chi‐square test for categorical variables and ANOVA for quantitative data.

**FIGURE 9:**
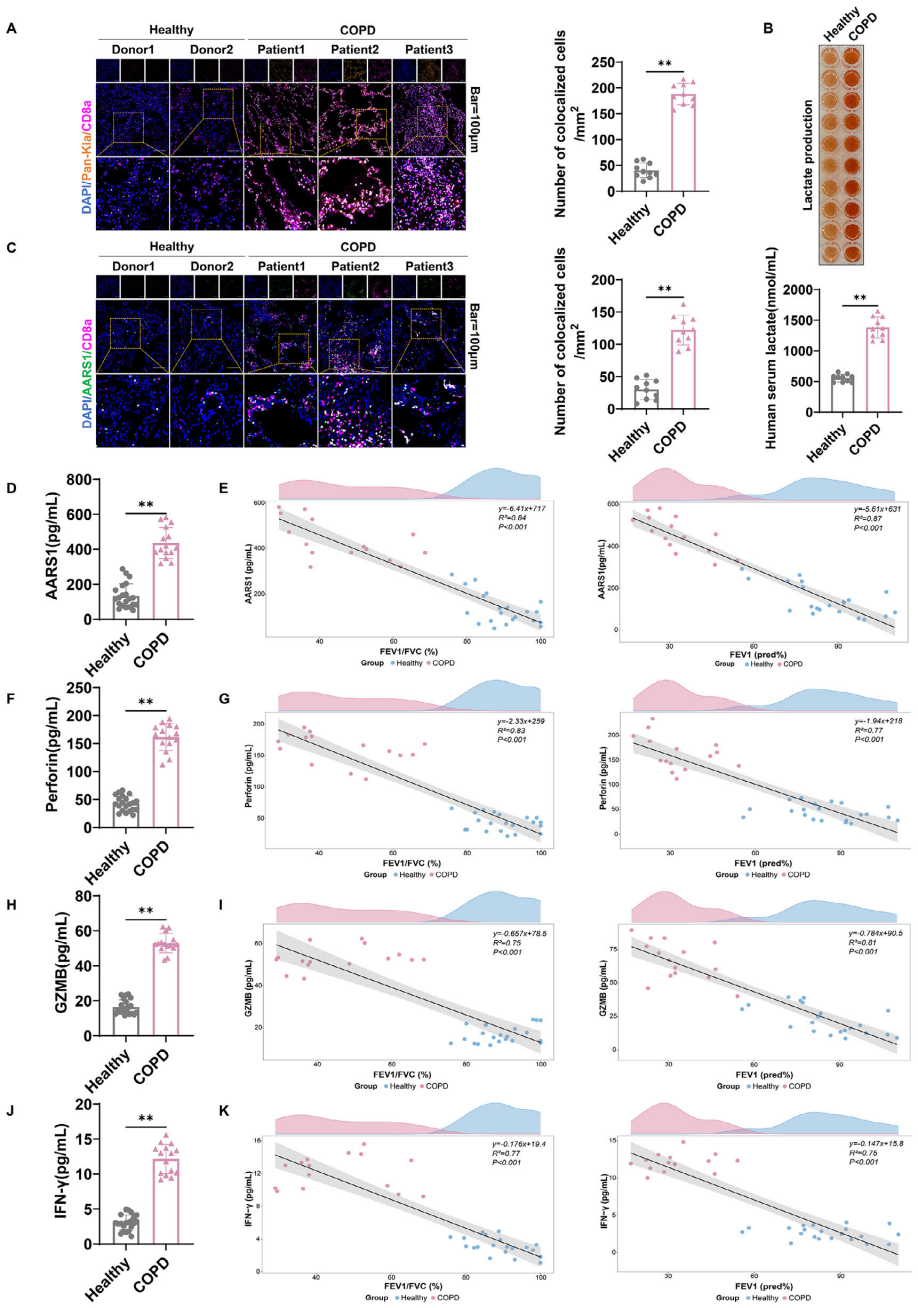
Elevated serum biomarker levels are related to COPD progression. (A) Representative Pan‐Kla/CD8a co‐localization immunostaining images (left) and corresponding quantitative analysis (right) of lungs from healthy donors (*n* = 10, biological replicate) and COPD patients (*n* = 10, biological replicate) (scale bar, 100 µm). (B) Representative images (top) and quantitative analysis (bottom) of lactate in serum of healthy (*n* = 10, biological replicates) and COPD patients (*n* = 10, biological replicates). (C) Representative AARS1/CD8a co‐localization immunostaining images (left) and corresponding quantitative analysis (right) of lungs from healthy donors (*n* = 10, biological replicate) and COPD patients (*n* = 10, biological replicate) (scale bar, 100 µm). (D‐K) The levels of AARS1, Perforin, GZMB, and IFN‐γ in serum of healthy (*n* = 21, biological replicates) and COPD patients (*n* = 15, biological replicates) were quantified by ELISA, and correlative regression analysis of serum Perforin, GZMB, and IFN‐γ with FEV1/FVC (%) or predicted FEV1 (%). Data are presented as means ± SEM. ^**^ *p* < 0.01, by Student's unpaired *t*‐test.

## 3 Discussion

Although we have learned a great deal about the characteristic pathological features of emphysema, including loss of elastic fibers in alveolar walls and abnormal expansion of alveolar spaces, effective treatment methods remain limited. (GlaxoSmithKline and Bayer outside the Submitted Work. Dr Cho Reported Receiving Grants From GlaxoSmithKline and Bayer, Consulting Fees From Genentech and AstraZeneca, and Speaking Fees From Illumina outside the Submitted Work. Professor Tobin and Professor Wain Reported Receiving Grants From GlaxoSmithKline and Orion outside the Submitted Work. Dr Beaty reported receiving grants from the National Heart, Lung, and Blood Institute (NHLBI) during the conduct of the study. No other disclosures were reported. See Ref. [27]). Clinical studies reveal that the numbers of CD8^+^T cells infiltrating lungs are higher in emphysema patients than in healthy individuals, primarily congregating in areas of alveolar destruction [5, 28]. The role of CD8^+^T cells in regulating alveolar microenvironmental homeostasis raises a question about their latent functional properties. To investigate this, we utilized human samples and various model systems, including mouse models of emphysema, and primary CD8^+^T cells and lung organoids. Herein, we found that CD8^+^T cell activation was enriched in the lungs derived from COPD patients as well as in experimental emphysema mouse models, and we demonstrated the relevance of AARS1‐mediated lactylation in CD8^+^T cell activation. Furthermore, studies with mouse lung organoids revealed that CD8^+^T cell activation induces alveolar epithelial cell apoptosis and suppresses alveolar regeneration, thereby contributing to the pathogenesis of emphysema.

CD8^+^T cell activation and functional differentiation are associated with metabolic pathways leading to lactate production [21]. In the present work, we established that reprogramming of endogenous metabolic glycolysis drives lactylation in CSE‐treated CD8^+^T cells. Analysis of our scRNA‐seq data revealed the elevation of AARS1, a lactyltransferase, in CD8^+^T cells of patients with COPD. Additionally, we modulated glycolysis and AARS1 to explore their roles in activated CD8^+^T cells from the mouse model of emphysema. In view of the roles of glycolysis in immune reprogramming and epithelial dysfunction [29], this glycolysis‐dependent CD8^+^T cell activation may contribute to the pathological changes in emphysema. In the emphysema model, inhibition of glycolysis with DCA reduced the frequency of activated CD8^+^T cells and alleviated emphysema pathological changes in mouse lungs. The role of AARS1, mediating lactylation in CD8^+^T cells, contributes to CD8^+^T cell activation.

Nevertheless, there are inherent limitations of our in vivo therapeutic approaches. As a pan‐glycolytic inhibitor delivered via intraperitoneal injection, DCA lacks cell‐type selectivity and broadly suppresses glycolysis in nearly all pulmonary cell populations, including alveolar epithelial cells, macrophages, and neutrophils, all of which are participants in COPD pathogenesis. Therefore, the pulmonary protective effect of DCA cannot be attributed solely to the suppression of CD8^+^T cell activation, and we cannot rule out the protective contributions from other structural and inflammatory cells. Similarly, intranasally administered _β_LNPs@AARS1‑siRNA has no cell‐specific targeting property, so AARS1 is also knocked down in various lung cells apart from CD8^+^T cells. The observed therapeutic efficacy may thus originate from multiple pulmonary cell subsets rather than CD8^+^T cells alone. To clarify the cell‐autonomous function of AARS1 specifically in CD8^+^T cells, future research can construct CD8^+^T cell‐specific AARS1 conditional knockout mice or adopt CD8‐targeted nanoparticle delivery systems to avoid off‐target interference. The current intervention still reflects the overall therapeutic effect of systemic AARS1 inhibition in emphysema mice. Although these data demonstrate that targeting of AARS1 affects lactylation in CD8^+^T cells, we acknowledge potential non‐enzymatic effects on lactylation, which may constitute one of several regulatory pathways in CD8^+^T cell activation. Moreover, intranasal LNPs‐mediated AARS1 silencing represents a promising therapeutic strategy to limit excessive CD8^+^T cell‐mediated pulmonary cytotoxic injury and to slow emphysema progression. However, large‐scale, multicenter prospective clinical cohorts are required to verify the diagnostic efficiency of these two biomarkers, and systematic preclinical safety evaluation is essential before translating this nanoparticle therapeutic regimen into clinical application.

Our study explored the relationships between lactylation and protein degradation. An overlap analysis of lactylated proteins versus differentially expressed proteins indicated that lactylation influences protein levels in CD8^+^T cells. Protein lactylation, a posttranslational modification that mediates the stability and function of proteins, is a potential mechanism to account for this observation [30, 31]. In non‐small cell lung cancer, lactylation of APOC2 at the K70 site stabilizes protein levels by inhibiting its ubiquitination [32]. In CSE‐treated CD8^+^T cells, the elevation of AARS1 protein is concomitant with increased RUNX3 levels due to the inhibition of RUNX3 degradation. The UPS and the autophagy‐lysosomal pathway are the two degradation systems maintaining cellular protein homeostasis [33]. The accumulation of RUNX3 in CD8^+^T cells upon exposure to CQ but not to MG132 suggests that RUNX3 degradation is predominantly mediated via the autophagic pathway. Our data demonstrate that lactylation at the K193 site of RUNX3 stabilizes the protein by blocking its recognition and engulfment by autophagosomes.

RUNX3, a member of the Runt domain‐containing transcription factor family, has regulatory roles in immune cell activation, differentiation, and tissue homeostasis [34]. In cancers, RUNX3 is a regulator of CD8^+^T cell effector function. In Hodgkin's lymphoma, for instance, DNA methylation reprogramming at the RUNX3 locus enhances CD8^+^T cell infiltration into tumors and amplifies their cytotoxic potential, positioning such methylation as a mediator of antitumor immune responses [35]. Our data reveal that CSE increases RUNX3 protein abundance in CD8^+^T cells, and knockdown of RUNX3 reverses this activation. This reversal confirms that RUNX3 serves as a hub for transducing smoke‐induced cellular signals into pathogenic CD8^+^T cell responses. Thus, these observations extend the established function of RUNX3, delineating its previously unrecognized role in driving aberrant CD8^+^T cell activation during the development of smoking‐related emphysema.

In summary, our findings demonstrate that the activation of CD8^+^T cells exerts a function in CS‐induced emphysema. When CD8^+^T cells undergo CS exposure, elevated AARS1 levels in these cells transfer a lactyl group to RUNX3 at the K193 site. This increased lactylation modifies RUNX3, inhibiting its autolysosomal degradation. The elevated levels of RUNX3 promote CD8^+^T cell activation. Consequently, activated CD8^+^T cells secrete Perforin, GZMB, and IFN‐γ, inducing alveolar epithelial cell apoptosis and inhibiting alveolar regeneration, driving the progression of emphysema. Additionally, serum levels of lactate and AARS1 are elevated in COPD smokers. Therefore, both lactate and AARS1 could function as biomarkers for diagnosis and therapy in CS‐induced emphysema.

## 4 Methods

### 4.1 Human Samples

All human lung tissue samples utilized in this research were obtained from individuals undergoing surgical lobectomy for benign pulmonary nodules or lung transplantation at Wuxi People's Hospital, affiliated with Nanjing Medical University. This research received formal approval from the Ethics Committee of Nanjing Medical University (Approval No.: 2021–130). All samples were harvested after obtaining signed, informed consent from donors and rendered anonymous prior to laboratory analysis. The clinical diagnosis of COPD complied fully with the criteria outlined in the Global Strategy for the Diagnosis, Management, and Treatment of Chronic Obstructive Pulmonary Disease. Serum samples for ELISA experiments were collected from a cohort of healthy controls and COPD patients. Clinical and pathological profiles of the study participants are provided in Table 1 and Table S1.

### 4.2 Animals

Six‐week‐old male BALB/c mice were acquired from the Laboratory Animal Center of Nanjing Medical University and maintained in a specific pathogen‐free (SPF) facility at the Jiangsu Provincial Center for the Safety Evaluation of Drugs, Pesticides, and Veterinary Drugs. The animal experimental protocol underwent rigorous review and received formal approval from the Institutional Animal Care and Use Committee of Nanjing Medical University (IACUC‐2512085). Mice were placed in SPF chambers under strictly controlled environmental conditions: a stable temperature of 23±3°C, relative humidity ranging from 40% to 70%, and a standardized 12‐h light‐dark cycle. Drawing on published literature and our team's previous experimental data, we determined the optimal CS concentration for murine models and established a CS‐induced emphysema model. Yoshida and colleagues adopted a whole‐body CS exposure system to induce emphysema in 6‐8‐week‐old mice; animals were exposed to smoke generated by 3R4F reference cigarettes (total suspended particulates: 200 mg/m^3^) on five consecutive days per week over a six‐month period [36]. Each group of 10 mice was exposed to smoke from 5 3R4F reference cigarettes 2 times daily, with 30‐min smoke‐free intervals between exposures, 5 days per week for 24 weeks. Their lungs exhibited alveolar cavity enlargement and alveolar wall destruction [37]. In our earlier investigation, we subjected mice with CS‐induced emphysema to systemic exposure of 300 mg/m^3^ total particulate matter (TPM). The treatment schedule covered 16 consecutive weeks, with two 4‐h spaced exposure sessions implemented five days every week [37]. Furthermore, we computed matched environmental tobacco smoke (ETS) exposure doses for humans and mice by applying previously published dose conversion models for ETS exposure [38, 39]. Mice were exposed to CS concentrations of 0, 200, or 300 mg/m^3^, corresponding to human exposure levels of non‐smokers, moderate smokers (0.5–1 packs/day), and heavy smokers (>1 packs/day), respectively [40]. The present research adopted a previously reported exposure protocol for establishing a mouse emphysema model [41]. In brief, we placed the mice in a whole‐body CS exposure system supplied by Beijing Huironghe Technology Co., Ltd., China. The system delivered TPM to the experimental animals at 200 or 300 mg/m^3^, which was obtained from University of Kentucky 3R4F research cigarettes. Exposure was administered twice daily for 1 h per session, separated by a 4‐h smoke‐free window, and conducted 5 days each week for 24 consecutive weeks. Age‐paired mice not exposed to CS in a similar environment served as controls. Our results showed that CS elicited alveolar wall disruption in mouse lungs; the increase in mean linear intercept represented a pathological feature associated with the development of smoking‐related emphysema. (Figure 2A,B).

### 4.3 Isolation and Culture of CD8^+^T Cells

Primary CD8^+^T cells from the spleen were isolated using an established experimental procedure [42]. Male BALB/c mice aged 8 weeks were utilized and euthanized with approval of the NJMU Animal Ethics Committee. Primary CD8^+^T cells were isolated from the spleens of 8‐week‐old mice using Mouse CD8^+^T Cell Isolation Kits (Vazyme, CS103‐01) according to the manufacturer's instructions. Subsequently, ImmunoCult Mouse T Cell Activator (STEMCELL, 100–1572) diluted 1:100 was used to activate CD8^+^T cells for 20 h, followed by treatment with 10 µg/mL brefeldin A for 4 h. We homogenized spleen samples to obtain cell suspensions, filtered the mixtures through a 70‐µm strainer, and then centrifuged the preparations at a rotational speed of 1500 rpm for 5 min. We then suspended the cells in 1 mL of red blood cell lysis buffer (Solarbio, R1010) and incubated them for 5 min, after which we repeated centrifugation at 1500 rpm for another 5 min. After centrifugation and filtration, the cells were placed in medium containing 10% fetal bovine serum (Life Technologies/Gibco, 10099141C), 2 mm L‐glutamine (STEMCELL, 07100), 50 µM β‐mercaptoethanol (MCE, HY‐Y0326), and 0.1% penicillin‐streptomycin (Beyotime, C0222). Additionally, 10 ng/mL mouse recombinant IL‐2 and 10 nmol/L OVA‐N4 were added to the medium. Cells were cultured for 3 days at 37°C under 5% CO_2_ conditions prior to subsequent experiments. All procedures were performed under sterile conditions. We validated the purity of primary CD8^+^T cell cultures through flow cytometry analysis, for which the percentages of target cells were above 90% (Figure S5A,B).

Based on existing studies and the correlation between exposure levels in human subjects and corresponding cell culture conditions, we derived the CSE concentrations to be used for our cell culture system. As previously mentioned, there is an increase in lymphocyte numbers (primarily CD8^+^T cells) in lungs from COPD patients [43]. Further, the extent of alveolar destruction and decline in lung function positively correlates with the degree of CD8^+^T cell infiltration into the lungs of COPD patients [44], and exposure to CS reduces the proportion of CD8^+^T cells in the lungs of COPD patients and reduces the proportion of CD4^+^T cells [45]. Our previous work demonstrated that, for cell culture systems, 1%, 2%, and 4% CSE promote emphysema progression via the induction of macrophage M2 polarization [24]. In the present study, CSE‐exposed primary CD8^+^T cells exhibited elevated activation rates in a dose‐response relationship (Figure S5C). Furthermore, CSE concentrations ranging from 2.5% to 10% correspond roughly to daily smoking exposures equivalent to 0.5–2 cigarette packs [46]. Based on prior related studies, immune cell treatments with 0%, 1%, 2%, or 4% CSE, respectively, mirror human smoking exposure intensities of no exposure, light smoking (0.5 packs/day), moderate smoking (0.5–1 packs/day), and heavy smoking (>1 packs/day) ([40]). Building on this, we assessed CD8^+^T cell activity following exposure to various CSE concentrations (Figure S6). Thus, we treated primary CD8^+^T cells with 0%, 1%, 2%, or 4% CSE.

### 4.4 Establishment and Utilization of a CD8^+^T Cell‐Lung Organoid Co‐Culture Model

CD8^+^T cells treated with 0% or 4% CSE for 48 h were seeded into co‐culture chambers, and the medium was replaced with fresh medium containing 10 nm DCA or 50 nm AARS1 siRNA (Table S2). After 6 h, the CD8^+^T cells were co‐cultured with lung organoids for 24 h, and the average lung organoid diameter (organoid size) was measured via NIS‐Elements software under an optical microscope.

### 4.5 Preparation of _β_lipid Nanoparticles@AARS1‐siRNA (_β_LNPs@AARS1‐siRNA)

Lipid nanoparticles (LNPs) were prepared using lipid components as follows: Dlin‐MC3‐DMA (an ionizable cationic lipid, 50 mol%), DSPC (1,2‐distearoyl‐sn‐glycero‐3‐phosphocholine, 10 mol%), cholesterol (38.5 mol%), and DMG‐PEG 2000 (1,2‐dimyristoyl‐rac‐glycerol‐3‐methoxy‐polyethylene glycol‐2000, 1.5 mol%). The chemically modified PEG lipid, DSPE‐PEG 2000‐maleimide (Merck, 880224P), was added to the lipid component at a 1:3 molar ratio of DSPE‐PEG 2k‐maleimide to DMG‐PEG 2000. An optimized LNP formulation (designated as X% _β_LNPs, where X% represents the molar ratio of β‐sitosterol to total lipids) was adapted from a previously reported protocol [26] with a minor modification: cholesterol was replaced with an equimolar amount of β‐sitosterol to improve siRNA delivery efficiency into immune cells. All lipid reagents were purchased from Avanti Polar Lipids (Alabama, USA) with a purity ≥99%. Lipids were co‐dissolved in 1 mL dichloromethane/methanol mixture (1:1, v/v), and the organic solvent was removed via rotary evaporation to yield a homogeneous lipid film. Subsequently, 1 mL of sterile phosphate‐buffered saline (PBS) containing AARS1 siRNA was added, and the mixture was incubated at 60°C for 1.5 h to hydrate the lipid film and encapsulate the siRNA. Encapsulation efficiency (EE) was quantified using either high‐performance liquid chromatography (HPLC) or fluorescence spectrophotometry, with a target EE ≥ 80%. After hydration, the liposome suspension was transferred to a liposome extruder and sequentially extruded 10 times through 400‐nm, 200‐nm, and 100‐nm polycarbonate membranes at 37°C, yielding uniform LNPs with particle sizes < 200 nm. Finally, the LNP suspension was purified using a Sephadex G‐25 column to remove free siRNA and unbound lipids.

### 4.6 Statistical Analysis

Experimental data were presented as means ± SD or SEM, with all values derived from no fewer than three independent experiments. Statistical analyses were conducted using GraphPad Prism 10 software (GraphPad Software Inc., San Diego, CA), where differences with *p* < 0.05 or *p* < 0.01 were defined as statistically significant. Statistical differences were determined by Student's unpaired t‐test or by one‐way ANOVA with Bonferroni correction. Calculations between two parameters were performed using regression analysis.

## Author Contributions

Conceptualization, **Ying Zhu**, **Haibo Xia**, and **Qizhan Liu**; methodology, Ying Zhu, **Jiaheng Lin**, **Yifan Li**, **Qiaoqiao Zhou**, Haibo Xia, and Qizhan Liu; investigation, Ying Zhu; resources, Ying Zhu, Jiaheng Lin, **Yan Wu**, Haibo Xia, and Qizhan Liu; writing – original draft, Ying Zhu; writing – review & editing, Ying Zhu, Jiaheng Lin, Yifan Li, Qiaoqiao Zhou, **Min Yu**, **Cheng Cheng**, **Kang Chang**, **Dang Lin**, **Tao Bian**, Haibo Xia and Qizhan Liu; supervision, Qizhan Liu; funding acquisition, Ying Zhu, Yan Wu, **Lu Lu**, Haibo Xia and Qizhan Liu.

## Funding

This work was funded by the National Natural Science Foundation of China (82574132, 82504445); the Basic Research Program of Jiangsu (BK20250307, BK20240523); the Jiangsu Funding program for Excellent Postdoctoral Talent (2025ZB419); the China Postdoctoral Science Foundation (2025M770751); the Jiangsu Postgraduate Scientific Research Innovation Plan (SJCX24_0805); and the Medical Key Strategic Project of Wuxi Health Commission (2025TH‐GD‐WY‐2025).

## Ethics Declarations

The ethics committee of Nanjing Medical University approved all human research protocols (number: 2021–130). All animal experiments were reviewed and authorized by the Animal Ethics Committee of Nanjing Medical University (IACUC‐2512085).

## Conflicts of Interest

The authors declare no conflicts of interest.

## Supporting information




**Supporting File**: advs76949‐sup‐0001‐SuppMat.docx.

## Data Availability

The data that support the findings of this study are available on request from the corresponding author. The data are not publicly available due to privacy or ethical restrictions.
