# Supplementary material for: AARS1‐Mediated RUNX3 K193 Lactylation Enhances the Activation and Cytotoxicity of CD8+T Cells in Smoking‐Related Emphysema
Source: Adv Sci (Weinh). 2026 Aug 6;13(60):e76949. doi: 10.1002/advs.76949 (PMC13445330; doi:10.1002/advs.76949)
Supplement: Supplementary file 1 [file ADVS-13-e76949-s001.docx]

**Supplementary information**

**Supplementary methods**

***Cigarette smoke extract (CSE)***

CSE was prepared according to previously reported methods [1]. Briefly, 3R4F research cigarettes (University of Kentucky, USA) were connected to one end of a vacuum pump, with the other end connected to a flask containing 10 mL of RPMI-1640 medium or Dulbecco's Modified Eagle Medium at 37°C. Each cigarette was then drawn at a constant rate for 5 minutes. After adjusting the pH to 7.4, the CSE solution was sterilized by filtration through a 22 µm pore filter (Schleicher & Schuell GmbH, Dassel, Germany). For quality control, the solution was monitored spectrophotometrically between 320 and 540 nm. Solutions were considered acceptable when ΔOD ranged between 0.9 and 1.2. The resulting solution was considered as 100% CSE and was prepared one hour prior to each experiment.

***Cell viability***

Cells were seeded at a density of 1×10⁴ cells per well in a 96-well microplate. Cell viability was determined using CCK8 assay kits (MCE, Product No. HY-K0301), with the procedure adhering to the manufacturer's instructions.

***Establishment and intervention of a CD8⁺T cell-MLE-12 cell co-culture model***

CD8^+^T cells were seeded into co-culture chambers. After exposing CD8^+^T cells to 0%, 1%, 2%, or 4% CSE for 48 hours, fresh medium was added. Subsequently, MLE-12 cells were seeded into six-well plates, and the co-culture chambers were transferred onto six-well plates to establish the CD8^+^T cell-MLE-12 cell co-culture model. CD8^+^T cells were seeded into co-culture chambers and treated with fresh medium containing 10 nM sodium dichloroacetate (DCA) (MCE, 2156-56-1) or 50 nM AARS1 siRNA (Table S2) for 48 hours. The cells were then co-cultured with MLE-12 cells for an additional 24 hours, after which MLE-12 cells were harvested for subsequent analysis.

***Construction and culture of mouse lung organoids***

Fresh lung tissue was rinsed several times with cold sterile PBS containing 0.1% penicillin-streptomycin. Subsequently, the lung tissue was cut into approximately 1mm³ chunks and suspended in 5ml organoid primary digestion buffer. Each tissue fragment was digested in DMEM/F12 medium at 37°C for approximately 1 hour, with digestion terminated when > 80% of single cells had dissociated from the tissue. The cell suspension was passed through a 100-μm pore filter and centrifuged at 1500 rpm for 5 minutes. Subsequently, the cells were suspended in 2 mL of red blood cell lysis buffer (100 μM EDTA, 10 mM KHCO₃, and 155 mM NH₄Cl) for 8 minutes. The cell pellet was collected, suspended in Matrigel (Corning, USA), and seeded onto pre-warmed 24-well tissue culture plates at 30-50 μL per well. The 24-well plates were transferred to a 37°C incubator for 10 minutes. After Matrigel polymerization, 1 mL lung organoid culture medium (DMEM/F12 medium containing 5% FBS, 4 mM l-glutamine, 10 µg/ml insulin, 5 µg/ml ITS, 0.1 µg/ml cholera toxin, 25 ng/ml HGF, and 25 ng/ml FGF), supplemented with 0.01 µg/ml retinoic acid (RA) medium at 37°C was added. The liquid was changed twice a week. Using NIS-Elements software, the average diameters of organoids (organoid size) were measured under an optical microscope.

***Small interfering RNA, plasmids, and transfection***

Transfection of primary CD8⁺T cells with siRNA synthesized by Sangon Biotech (Shanghai) (Table S2) was performed using the Lonza 4D-Nucleofector™ system and the P3 Primary Cell 4D-Nucleofector™ X Kit (Lonza, V4XP-3024) with the primary T cell-specific program (“DZ-100” for murine cells) to target either RUNX3 or AARS1. Primary CD8⁺T cells were transfected with pcDNA3.1-RUNX3-3×Flag overexpression plasmids and pcDNA3.1 empty vector controls (Table S2) constructed by Corues Biotechnology (Nanjing), using the same electroporation system and protocol. Following transfection, cells were treated with 0%, 1%, 2%, or 4% CSE for 48 hours, or co-cultured with MLE-12 cells for an additional 24 hours before collection. Cells were subsequently harvested for analysis.

***Point mutation experiment***

We constructed Flag-RUNX3-WT as template DNA using WZ Bioscience (Shandong). We constructed Flag-RUNX3-K88R, Flag-RUNX3-K95R, Flag-RUNX3-K130R, and Flag-RUNX3-K193R point mutation plasmids. Following RUNX3 siRNA-mediated knockdown of RUNX3 levels in CD8^+^ T cells, 2 μg of Flag-RUNX3-WT, Flag-RUNX3-K88R, Flag-RUNX3-K95R, Flag-RUNX3-K130R, or Flag-RUNX3-K193R point mutation plasmids were transferred into CD8^+^T cells using an electroporator. Cells were then treated with either 0% or 4% CSE for 48 hours before collection, or co-cultured with MLE-12 cells for 24 hours before collection. Cells were subsequently harvested for analysis.

***Protein extraction and western blots (WB)***

Total proteins were extracted from cells, lung organoids, or lung tissues using RIPA lysis buffer (Beyotime, P0013B); the procedures were as follows: For lung tissues (0.5 g), samples were homogenized in 200 µl RIPA lysis buffer using a tissue homogenizer, followed by sonication and centrifugation at 4°C and 12,000 × g for 15 minutes. For adherent cells cultured in 100-mm diameter cell culture dishes (Thermo Fisher Scientific, 150466), the culture medium was aspirated, and 100 µl RIPA lysis buffer was added to the dishes. For lung organoids, residual Matrigel and culture medium were removed by washing with cooled PBS (pH 7.4) three times (4°C, 300 × g for 5 minutes each), and the organoids were then suspended in 100 µl RIPA lysis buffer, homogenized, and incubated on ice for 30 minutes. All samples were centrifuged at 4°C and 12,000 × g for 15 minutes, and the supernatants were collected. Protein concentrations were determined using BCA protein assay kits (Beyotime, P0010S) according to the manufacturer’s instructions. Samples were run on a 10% SDS-PAGE gel and transferred to nitrocellulose membranes. The membranes were blocked with 5% BSA in Tris-buffered saline with Tween 20 (TBST) at 37°C for 1 hour, followed by overnight incubation with primary antibody at 4°C. The membranes were washed three times with TBST for 30 minutes each, then incubated for 1 hour with anti-immunoglobulin horseradish peroxidase-conjugated antibody (Beijing Zhongshan Jinqiao Bio-Tech Co., Ltd., China) diluted 1:10,000. The preparations were washed three times with TBST for 30 minutes each, then evaluated with chemiluminescent reagent (Tanon, 180-5001). Western blots were replicated at least three times and quantified using ImageJ. Primary antibodies were α-Tubulin (Proteintech, 80762-1-RR), Axin2 (Proteintech, 20540-1-AP), β-catenin (Abcam, ab32572), Bax (Starter, SDT-338-76), Bcl-2 (Starter, SDT-005-24), Perforin (Proteintech, 14580-1-AP), GZMB (Proteintech, 13588-1-AP), IFN-γ (Abclonal, A27845), ENO-1 (Proteintech, 11204-1-AP), HK2 (Proteintech, 22029-1-AP), LDHA (Proteintech, 19987-1-AP), Lat (ServiceBio, GB111123-100), PCIF1(ServiceBio, GB114736-100), AlaRS (Proteintech, 67909-1-Ig), RUNX3 (Proteintech, 27099-1-AP), Pan-Kla(PTMBIO, PTM-1401RM), and DYKDDDDK tag (Proteintech, 66008-4-Ig).

***Immunoprecipitation (IP)***

We followed the instructions for Pierce Classic Magnetic Bead IP/Co-IP Kit (Thermo, 88804). Briefly, cells were harvested and suspended in an appropriate volume of Cell IP Lysis Buffer (containing protease inhibitors). Cells were lysed for 30 minutes on ice or at 4°C, centrifuged at 12,000g for 30 minutes, and the supernatant was removed. For WB analysis of a small amount of lysate, 1 μg of the corresponding antibody and Protein A/G beads (10-50 μL) were added to the cell lysate, then gently rocked. Following IP, the protein A/G beads were centrifuged at 3000g for 5 minutes at 4°C. The supernatant was aspirated, and the beads were washed 3-4 times with buffer. Finally, 15 μL of 2 × SDS loading buffer was added, and the preparations were placed in boiling water for 10 minutes.

***Cycloheximide (CHX), Chloroquine (CQ), and Z-Leu-Leu-Leu-al (MG132) chase assays***

After exposure of CD8^+^T cells to 0% or 4% CSE, cells were exposed to CHX (20 μg/mL, MedChemExpress, HY-12320) for 0, 8, 16, or 24 hours; to CQ (50 μM, MedChemExpress, HY-17589); or to MG132 (10 μM, MedChemExpress, HY-13259) for 24 hours. Total protein lysates were harvested and subjected to WB to determine the degradation rate of RUNX3.

***Reverse transcriptase and quantitative real-time PCR***

Total RNA was isolated from cultured cells and serum using Trizol reagent (Thermo Fisher Scientific, 15596026CN). Total RNA (1 µg) was transcribed into cDNA using HiScript II Q RT SuperMix (Vazyme Biotech, R222-01). Real-time qPCR was performed using AceQ® qPCR SYBR® Green Master Mix (Vazyme Biotech, Q111-02) on a LightCycler 480 II system or Quant Studio 5. α-Tubulin mRNA served as the internal control. PCR primer sequences listed in Table S3 were obtained from Sangon Biotech, Shanghai, China.

***Flow cytometry analysis***

For flow cytometric analysis, CD8^+^T cells were washed twice with cold PBS to exclude dead cells, then stained with Fix & Viable Reagent Kits (Biolegend, 423105) at room temperature for 20 minutes. Subsequently, cells were incubated at 4°C for 20 minutes with the following fluorescently labeled surface marker-specific antibodies: anti-mouse CD45 (BD, 563891), anti-mouse Ly6G (BD, 563979), anti-mouse CD3 (BD, 553061), and anti-mouse CD8 (BD, 561109). For intracellular staining, cells were fixed and permeabilized with Fix Buffer (Bio-Rad, 420801) for 20 minutes and washed with Intracellular Staining Permeabilized Wash Buffer (Bio-Rad, 421002). Cells were then stained with anti-mouse IFN-γ (BD, 1554413). Finally, cells were suspended in Cell Staining Buffer (Biolegend, 4201201) and analyzed on a FACS Symphony SORP instrument (BD Biosciences, USA). Data were analyzed using FlowJo V10 software (Tree Star, USA).

***ELISA***

CD8^+^T cell supernatants and human serum samples were collected. We analyzed Perforin (Elabscience, E-EL-M0890, E-EL-H1123), GZMB (Elabscience, E-EL-M0594, E-EL-H1617), IFN-γ (Elabscience, E-EL-M0048, E-EL-H0108), and AARS1 (Yuhengfeng Bio, Abx585477). Standard curves were plotted based on results for standard products, and corresponding sample results were calculated. All samples underwent duplicate testing.

***Measurement of lactate levels***

Lactate levels in cell supernatants, mouse lung tissues, and human serum were quantified using L-Lactic Acid Assay Kits (Beyotime, S0208S) according to the instructions. Cell supernatants were diluted 10-fold, mouse lung tissue samples 100-fold, and serum samples 500-fold. Lysis of mouse lung tissue was performed with RIPA buffer, with the supernatant used for lactate measurement. Lactate content in mouse lung tissue was normalized by tissue weight.

***Seahorse analysis (ECAR&OCR)***

Extracellular acidification rate (ECAR) and oxygen consumption rate (OCR) were assessed using a Seahorse XFe96 Flux Analyzer equipped with Mito Stress Test Kit (Seahorse Biosciences, Agilent Technologies, USA). CD8^+^T cells were seeded at a density of 2000 cells per well in XF96 plates. After 48-hour treatment with 0, 1, 2, or 4% CSE, test compounds were applied: ECAR: 10 mM glucose (Sigma-Aldrich), 1.5 μM oligomycin (Selleck), 200 mM 2-DG (Sigma-Aldrich); OCR: 1.5 μM oligomycin (Selleck), 1.0 μM carbonyl cyanide p-trifluoromethoxyphenylhydrazine (Sigma-Aldrich), and 0.5 μM rotenone/antimycin A (Sigma-Aldrich). ECAR and OCR measurements were averaged and normalized per well based on cell counts.

***Immunofluorescence (IF)***

After centrifuging CD8^+^T cells (1200 × g, 5 minutes), pellets were collected, and dispersed onto poly-L-lysine-coated slides (approximately 2×10⁴ cells per slide). These were incubated at room temperature for 30 minutes to allow cells to settle and adhere to the slides. The cells were fixed on slides with 4% paraformaldehyde at room temperature for 15 minutes, then washed three times with PBS (5 minutes each wash). The cells were permeabilized with 0.1% Triton X-100 in PBS at room temperature for 10 minutes, followed by three additional PBS washes (5 minutes each). They were blocked with 5% BSA-containing blocking solution at room temperature for 1 hour, and the blocking solution was discarded. After blocking, the preparations were incubated with primary antibodies: RUNX3 antibody (Proteintech, 27099-1-AP), LC3B antibody (ServiceBio, GB113801-100), or DYKDDDDK tag (Proteintech, 66008-4-Ig). Slides were placed in a humidified chamber and incubated overnight at 4°C. The next day, the slides were washed three times with PBS containing 0.05% Tween 20 (PBST) for 5 minutes each to remove unbound primary antibodies. Secondary antibodies were applied: Cy3 anti-mouse IgG (Beyotime, A0521), or FITC anti-rabbit IgG (Beyotime, A0562). They were incubated at room temperature in the dark for 1 hour. Then they were washed three times with PBST (5 minutes each), followed by a final wash with PBS to remove Tween 20. To visualize cell nuclei and prevent fluorescence quenching, a drop of DAPI fluorescent dye (Beyotime, C1006) was added to the cell region on the slide. The slides were covered with a coverslip (avoiding bubbles), and incubate at room temperature in the dark for 10 minutes to allow the mounting medium to solidify. The cells were visualized using an LSM 900 confocal microscope, and image analysis was performed with ZEN imaging software.

***Chromatin immunoprecipitation (ChIP) assays***

Chromatin immunoprecipitation (ChIP) experiments were accomplished with ChIP Assay Kits purchased from Thermo Fisher Scientific (catalog No. 26156). In brief, 1 × 10⁶ CD8⁺T cells were fixed via formaldehyde crosslinking at 37°C over a 10-minute incubation period. Following cell lysis, chromatin was fragmented through sonication in accordance with the manufacturer’s instructions. The soluble chromatin fractions were incubated overnight at 4°C under continuous rotational shaking together with target-specific primary antibodies to enrich protein-bound chromatin fragments; normal IgG was used as a negative control. Protein A/G magnetic beads were added to capture the immunoprecipitated chromatin-protein complexes with 60 minutes of incubation at 4°C. After multiple rounds of washing, the immunoprecipitated binding complexes were eluted, and the recovered, purified DNA was subjected to conventional ChIP-PCR or quantitative ChIP-qPCR amplification with primer pairs designed for the promoter sequences of target genes (Supplementary Table S4).

***LC-MS/MS analysis***

Immunoprecipitation (IP)-mass spectrometry (MS) analysis was performed on CD8⁺T cells exposed to 4% CSE for 48 hours, followed by transfection with RUNX3 siRNA or negative control (NC) siRNA for 6 hours. IP was conducted using an anti-RUNX3 antibody (Santa Cruz Biotechnology, (A-3): sc-376591), followed by protein separation via SDS-PAGE and visualization with Coomassie Brilliant Blue (CBB) staining. The gel band corresponding to the molecular weight of RUNX3 was excised, and the lysine lactylation (Kla) sites of RUNX3 were identified via MS analysis (QinglianBio, Beijing).

***Proteomics sequencing***

CD8⁺T cells were exposed to 0% or 4% CSE. Total proteins were extracted, quantified, and subjected to tryptic digestion to generate peptides. Peptides were analyzed by mass spectrometry (MS) to determine their mass-to-charge ratio (M/Z) and amino acid composition, from which amino acid sequences were deduced. Mass spectrometry data were compared against a database of known protein sequences to match peptide sequences with known proteins, thereby obtaining the primary structure of target proteins. Data processing and statistical analysis were performed using R software, with Student’s t-test used to assess significance. Proteins were considered significantly differentially expressed if they met the criteria of log₂ fold change (log₂FC) > 1.5 or < -1.5 and P < 0.05, compared to the 0% CSE control group.

***DCA treatment***

Six-week-old male BALB/c mice were randomly divided into four groups (n = 10 per group): normal control (exposed to clean air), CS exposure, CS exposure + DCA treatment, and CS exposure + vehicle control (normal saline). All mice were housed in a small animal whole-body dynamic exposure system. Mice in the CS-exposed groups (CS exposure, CS + DCA, and CS + vehicle control) were exposed to CS at a concentration of 300 mg/m³ TPM; the control group was exposed to clean air (exposure protocol as described previously). Following establishment of the emphysema model, mice in the CS + DCA group received intraperitoneal injections of DCA at a dose of 10 mg/kg twice weekly for 24 weeks. The vehicle control group received intraperitoneal injections of an equal volume of normal saline following the same schedule.

***_β_Lipid nanoparticles@AARS1-siRNA (_β_LNPs@AARS1-siRNA) treatment***

Six-week-old male BALB/c mice were randomly divided into four groups (n = 10 per group): normal control (exposed to clean air), CS exposure, CS exposure + _β_LNPs@AARS1-siRNA, and CS exposure + _β_LNPs@NC-siRNA (NC = negative control). All mice were housed in a small-animal, whole-body dynamic exposure system. Mice in the CS-exposed groups (CS exposure, CS + _β_LNPs@AARS1-siRNA, and CS + _β_LNPs@NC-siRNA) were exposed to CS at a concentration of 300 mg/m³ total TPM; the control group was exposed to clean air (exposure protocol as described previously). Following establishment of the emphysema model, mice in the CS + _β_LNPs@AARS1-siRNA group and CS + _β_LNPs@NC-siRNA group received intranasal instillation of _β_LNPs@AARS1-siRNA and _β_LNPs@NC-siRNA, respectively, at a dose of 10 μg _β_LNPs per mouse twice weekly for 24 weeks.

***Lung histopathology***

Mouse lung tissue was fixed in 4% paraformaldehyde for 24 hours, embedded in paraffin, and sectioned into 5-μm thick slices for hematoxylin and eosin (H&E) staining. Morphometric analysis was employed to quantify emphysema. Briefly, microscopic fields of view were selected randomly, and emphysematous changes in these fields were assessed using Image Pro Plus software. The commonly used metric for calculating alveolar space size, the mean linear intercept (chord) length (Lm), was calculated [2]. For IF staining, paraffin-embedded sections were deparaffinized, incubated with primary antibody overnight at 4°C, and incubated with secondary antibody for 1 hour at room temperature. Positive cells were randomly counted across different regions of the lung slide using confocal microscopy. Primary antibodies included: CD8 (Servicebio, GB11068), AARS1 (Proteintech, 17394-1-AP), SP-C (Abcam, ab90716) or Pan-Kla (PTMBIO, PTM-1401RM).

We quantified IF according to Lian et al. [3]. Using a 10× objective confocal microscope, positive lung cells were randomly counted from different angles. Twelve views per lung section were analyzed, determining the distribution of positive cells in human and mouse lung tissue based on the average cell count per 0.04 mm². Additionally, we normalized the total number of lung cells (DAPI^+^ cells) per lung field using ImageJ [4].

**References**

1. Xia H, Wu Y, Zhao J, et al. N6-Methyladenosine-modified circSAV1 triggers ferroptosis in COPD through recruiting YTHDF1 to facilitate the translation of IREB2. Cell death and differentiation. 2023 May;30(5):1293-1304. doi: 10.1038/s41418-023-01138-9. PubMed PMID: 36828914; PubMed Central PMCID: PMCPMC10154389. eng.

2. Knudsen L, Weibel ER, Gundersen HJ, et al. Assessment of air space size characteristics by intercept (chord) measurement: an accurate and efficient stereological approach. Journal of applied physiology (Bethesda, Md : 1985). 2010 Feb;108(2):412-21. doi: 10.1152/japplphysiol.01100.2009. PubMed PMID: 19959763; eng.

3. Lian Q, Zhang K, Zhang Z, et al. Differential effects of macrophage subtypes on SARS-CoV-2 infection in a human pluripotent stem cell-derived model. Nature communications. 2022 Apr 19;13(1):2028. doi: 10.1038/s41467-022-29731-5. PubMed PMID: 35440562; PubMed Central PMCID: PMCPMC9018716. eng.

4. Girardi F, Taleb A, Ebrahimi M, et al. TGFβ signaling curbs cell fusion and muscle regeneration. Nature communications. 2021 Feb 2;12(1):750. doi: 10.1038/s41467-020-20289-8. PubMed PMID: 33531466; PubMed Central PMCID: PMCPMC7854756. eng.

**Supplementary tables**

***Table S1. Clinical information for lung tissue donors.***

|  | **Gender** | **Age** | **Smoking history**  **(pack-years)** | **FEV_1_ (%) predicted** | **FEV_1_/FVC (%)** | **GOLD stages** |
| --- | --- | --- | --- | --- | --- | --- |
| Healthy-1 | male | 46 | - | 106.9 | 87.39 | - |
| Healthy-2 | male | 51 | - | 72.8 | 86.48 | - |
| Healthy-3 | male | 79 | - | 90.4 | 83.17 | - |
| Healthy-4 | male | 60 | - | 80.5 | 92.44 | - |
| Healthy-5 | male | 58 | - | 82.5 | 80.07 | - |
| Healthy-6 | male | 74 | - | 85.8 | 75.9 | - |
| Healthy-7 | male | 66 | - | 88.1 | 88.83 | - |
| Healthy-8 | male | 72 | - | 74.9 | 72.76 | - |
| Healthy-9 | male | 43 | - | 86.9 | 93.12 | - |
| Healthy-10 | female | 65 | - | 91.9 | 98.91 |  |
| COPD-1 | female | 63 | 20 | 62.01 | 70.46 | Ⅰ |
| COPD-2 | male | 73 | 100 | 66.2 | 73.36 | Ⅰ |
| COPD-3 | male | 77 | 20 | 42.3 | 49.01 | Ⅱ |
| COPD-4 | male | 67 | 50 | 47 | 51.74 | Ⅱ |
| COPD-5 | male | 64 | 20 | 50.2 | 48.63 | Ⅱ |
| COPD-6 | male | 77 | 40 | 42.01 | 50.46 | Ⅱ |
| COPD-7 | male | 63 | 80 | 44.1 | 41.99 | Ⅱ |
| COPD-8 | male | 79 | 40 | 26.6 | 29.49 | Ⅲ |
| COPD-9 | male | 71 | 150 | 30.7 | 35.4 | Ⅲ |
| COPD-10 | male | 66 | 40 | 17.3 | 28.04 | Ⅳ |

***Table S2. siRNA sequences used.***

| **Name** | **Sequence** |
| --- | --- |
| GPR81-Si1 | *5′-GAGCGTGAGGGAAAAAGACAAAA-3′* |
| GPR81-Si2 | *5′-GAGCTGCAACCCAGAAATGTACA-3′* |
| GPR81-Si3 | *5′-GACAGACTACTACCTCAGA-3′* |
| AARS1-Si1 | *5′-GGCGGGTGAGTATAGCTTCTTGT-3′* |
| AARS1-Si2 | *5′-GACCCTCGGGAAAAAAAGCAAGT-3′* |
| AARS1-Si3 | *5′-CTCTGATTGGATGGTGAGGTACG-3′* |
| RUNX3-Si1 | *5′-GGCTCGTCACCTTCGTGAATACC-3′* |
| RUNX3-Si2 | *5′-GCCATCAAGGTCACTGTGGATGG-3′* |
| RUNX3-Si3 | *5′-CGCTTCCGCTGTCATGAAGAACC-3′* |

***Table S3.*** ***Primer sequences used.***

| **Primer Name** | **Sequence** |
| --- | --- |
| Perforin | *F: 5’-CTGCCACTCGGTCAGAATG-3’*  *R: 5’- CGGAGGGTAGTCACATCCAT-3’* |
| GZMB | *F: 5’-TCTCGACCCTACATGGCCTTA-3’*  *R: 5’-TCCTGTTCTTTGATGTTGTGGG-3’* |
| IFN-γ | *F: 5’-GCCACGGCACAGTCATTGA-3’*  *R: 5’-TGCTGATGGCCTGATTGTCTT-3’* |
| GPR81 | *F：5′-CCGGTTCATCATGTGTGTGGTT-3′*  *R：5′-CTCTTCTGACCTCCGCGTCTCTTC-3′* |
| AARS1 | *F: 5’-ATGGATGCCACTTTAACAGCA-3’*  *R: 5’-TGGGTCGATTGTGTTTAGGAAGA-3’* |
| KAT7 | *F: 5’-CTCGAACTCCAACCGGAAATG-3’*  *R: 5’-TGTAGCTTTCGTGAAATCGACG-3’* |
| KAT8 | *F: 5’-ACCGTGGAGATCGGAGAAAC-3’*  *R: 5’-GCCTGTTAAAGCCCACATAGTGA-3’* |
| RUNX3 | *F: 5’-GACTCCTTCCCCAACTATACACC-3’*  *R: 5’-CGCTGTTCTCGCCCATCTT-3’* |
| Tubulin | *F: 5’-CCTAAACAGGTTGATAGGCCAAA-3’*  *R: 5’-CTCGCCTTCCACAGAATCCA-3’* |

***Table S4.*** ***Primer sequences used.***

| **Primer Name** | **Sequence** |
| --- | --- |
| Perforin-promoter | *F: 5’-GACAACTCGGGGGTTCATGT-3’*  *R: 5’-TTACCCCGTTCCCTGATGGT-3’* |
| GZMB-promoter | *F: 5’-GTCATGCTTGGTCCTGGTACA -3’*  *R: 5’-GGGTGGGGTTTGTAGCCTAT-3’* |
| IFN-γ-promoter | *F: 5’-AGCAAGGCGAAAAAGGATGC-3’*  *R: 5’-CAATCTGTCTGCAGTGGGGA-3’* |

**Supplementary figures and figure legends**

***Figure S1. Single-cell transcriptomic maps for lungs of COPD patients.***

(A) UMAP visualization of 20 lung cell lineages in lung tissues from healthy individuals and COPD patients. (B) Heatmap of representative genes for the 20 cell clusters. (C) Dotplot of representative genes for the 11 cell types. (D) Distribution of 11 cell types in healthy individuals and COPD patients. (E) UMAP visualization of scRNAseq from healthy individuals and COPD patients. (F) Box plots showing the proportions of distinct cell clusters in lungs of healthy individuals and COPD patients. (G) Box plots showing the proportions of 11 cell types in lung tissues of healthy individuals and COPD patients. (H) Pie charts of overall proportions of 11 cell types in healthy individuals and COPD patients.

***Figure S2. Single-cell transcriptomic profiles of T cells in lungs of COPD patients.***

(A) UMAP visualization of 9 T cell lineages in lung tissues from healthy individuals (n=3) and COPD patients (n=3). (B) Heatmap of representative genes for the 9 cell clusters. (C) Dotplot of representative genes for the 4 T cell types. (D) Distribution of 9 cell types in healthy individuals and COPD patients. (E) Box plots showing the proportions of 4 T cell types in lung tissues of healthy individuals and COPD patients. (F) Pie charts of overall proportions of 4 T cell types in healthy individuals and COPD patients.

***Figure S3. CD8^+^T cell activation in the lungs of COPD patients.***

(A and B) GSEA enrichment analysis of dysregulated mRNAs from T cells of healthy individuals and COPD patients. (C) Pseudo-time differentiation trajectory construction of CD8^+^T cells in healthy individuals and COPD patients. (D) Pseudo-time differentiation trajectories of CD8^+^T cells in healthy individuals and COPD patients.

***Figure S4. Single-cell transcriptomic maps of T cells in lungs of COPD patients.***

The analysis of scRNA-seq data from lung tissues of healthy individuals (n=3) and COPD patients (n=5) in the GSE173896 dataset. (A) UMAP visualization of scRNA-seq from healthy individuals and COPD patients. (B) Box plots showing the proportions distinct cell clusters in lungs of healthy individuals and COPD patients. (C) UMAP analysis of T cells from healthy individuals and COPD patients. (D) Box plots showing the proportions of distinct cell clusters in T cells of healthy individuals and COPD patients. KEGG (E) and GO (F) enrichment analysis of dysregulated mRNAs from T cells of healthy individuals and COPD patients. (G) Pseudo-time differentiation trajectories of CD8^+^T cells in healthy individuals and COPD patients. (H) Pseudo-time differentiation trajectories of distinct cell clusters in CD8^+^T cells.

***Figure S5.*** ***Establishment of a CS-induced emphysema mouse model.***

Six-week-old male BALB/c mice were exposed to cigarette smoke (CS) for 24 weeks prior to subsequent experiments, with total particulate matter (TPM) concentrations in smoke set at 0, 200, or 300 mg/m³. (A) Schematic diagram of the emphysema model. (B) Representative H&E-stained (left) and quantification (right) in lung sections of mice (scale bars: 100 μm and 20 μm). (C) Representative IF images (left) and quantification (right) of SP-C in lung tissues of mice (scale bar, 100 μm). (D) Representative immunoblot images (left) and quantification (right) of Axin2, β-catenin, and Bcl-2/Bax in lung tissues of mice. (E) Schematic workflow of flow cytometry analysis of lung tissue. Data are presented as means ± SEM, n = 6 biological replicate. ***P* < 0.01, by one-way ANOVA followed by Bonferroni correction.

***Figure S6. CSE triggers CD8^+^T cell activation and enhances its cell cytotoxicity in primary CD8^+^T cells of mice.***

Primary CD8^+^T cells were exposed to 0%, 1%, 2%, or 4% CSE for 48 hours. (A) Schematic of primary CD8^+^T cell isolation. (B) Flow cytometry of CD8^+^T cells. (C) Representative flow cytometry plots (left) and quantification (right) of IFN-γ^+^CD8^+^T cells. (D) Representative immunoblot images (left) and quantification (right) of Perforin, GZMB, and IFN-γ in CD8^+^T cells. (E) The levels of *Perforin*, *GZMB*, and *IFN-γ* in CD8^+^T cells measured by qRT-PCR. The levels of Perforin (F), GZMB (G), and IFN-γ (H) in the medium of CD8^+^T cells were quantified by ELISA assay. (I) Schematic diagram of the co-culture process between CD8^+^T cells and MLE-12 cells. (J) Representative immunoblot images (left) and quantification (right) of Axin2, β-catenin, and Bcl-2/Bax in MLE-12 cells. Data are presented as means ± SD, n = 3 independent experiments. ***P* < 0.01, by one-way ANOVA followed by Bonferroni correction.

***Figure S7. The effects of CSE on viability of CD8^+^T cells.***

Primary CD8^+^T cells were exposed to 0%, 0.5%, 1%, 2%, 4%, 8%, or 16% CSE for 48 hours. Quantification of cell viability by CCK8 assay. Data are presented as means ± SD, n = 3 independent experiments. ***P* < 0.01, by one-way ANOVA followed by Bonferroni correction.

***Figure S8. CD3/CD28 triggers CD8^+^T cell activation and enhances its cell cytotoxicity in primary CD8^+^T cells of mice.***

Primary CD8⁺T cells were assigned to four groups: control (CON, no anti-CD3/CD28 treatment), low-dose (LOW), normal-dose (NORMAL), and high-dose (HIGH) stimulation groups. Cells in the three activated groups were treated with gradient concentrations of plate-bound anti-CD3 combined with soluble anti-CD28 antibodies, followed by incubation for 48 h. The antibody concentrations for each group were set as follows: LOW group (plate-bound anti-CD3: 0.5 μg/mL, soluble anti-CD28: 0.25 μg/mL); NORMAL group (plate-bound anti-CD3: 2 μg/mL, soluble anti-CD28: 1 μg/mL); HIGH group (plate-bound anti-CD3: 4 μg/mL, soluble anti-CD28: 2 μg/mL). The levels of Perforin (A), GZMB (B), and IFN-γ (C) in the medium of CD8^+^T cells were quantified by ELISA assay. (D) Representative immunoblot images (left) and quantification (right) of Axin2, β-catenin, and Bcl-2/Bax in MLE-12 cells. Data are presented as means ± SD, n = 3 independent experiments. ***P* < 0.01, by one-way ANOVA followed by Bonferroni correction.

***Figure S9. GSK2837808A blocks the CSE-induced activation of CD8^+^T cells.***

GSK2837808A (30 µM) or vehicle control (0.1% DMSO, v/v) was added to CD8⁺T cells 6 hours before 48-hour exposure to 0% or 4% CSE. (A) Representative immunoblot images (left) and quantitative analysis (right) of Pan-Kla in CD8^+^T cells. (B) Representative flow cytometry plots (top) and quantification (bottom) of IFN-γ^+^CD8^+^T cells. (C) Representative immunoblot images (left) and quantification (right) of Perforin, GZMB, and IFN-γ in CD8^+^T cells. (D) The levels of *Perforin*, *GZMB*, and *IFN-γ* in CD8^+^T cells were measured by qRT-PCR. The levels of Perforin (E), GZMB (F), and IFN-γ (G) in the medium of CD8^+^T cells were quantified by ELISA. (H) Representative immunoblot images (left) and quantification (right) of Axin2, β-catenin, and Bcl-2/Bax in MLE-12 cells. Data are presented as means ± SD, n = 3 independent experiments. ***P* < 0.01, by one-way ANOVA followed by Bonferroni correction.

***Figure S10.*** ***Knockdown of GPR81 by siRNA has no effect on CSE-induced activation of CD8^+^T cells.***

GPR81 siRNA (50 µM) or NC siRNA (50 µM) was added to CD8⁺T cells 6 hours before 48-hour exposure to 0% or 4% CSE. (A) Representative immunoblots image (top) and quantification (bottom) of GPR81 in CD8^+^T cells. (B) The levels of *GPR81* in CD8^+^T cells measured by qRT-PCR. (C) Representative immunoblot images (left) and quantification (right) of GPR81 in CD8^+^T cells. (D) Representative flow cytometry plots (left) and quantification (right) of IFN-γ^+^CD8^+^T cells. Data are presented as means ± SD, n = 3 independent experiments. **P* < 0.05, ***P* < 0.01, by one-way ANOVA followed by Bonferroni correction. n.s., not significant.

***Figure S11. The levels of lactylation-related genes in CD8^+^T cells of COPD lungs.***

Gene array expression Series Matrix Files of CD8^+^T cells in the GSE167295, GSE173896 and GSE196638 databases were obtained from Gene Expression Omnibus data sets. Violin plots showing the expression of lactylation-related genes in CD8^+^T cells from the GSE167295 (A), GSE173896 (B), and GSE196638 datasets(C). Data are presented as means ± SEM. **P* < 0.05, ***P* < 0.01, *****P* < 0.0001, by Student’s unpaired t-test and each dot represents one individual subject (biological replicate). n.s., not significant.

***Figure S12. Knockdown of AARS1 reverses in CSE-treated CD8^+^T cell activation.*** Evaluation of AARS1 knockdown efficiency among three siRNAs in CD8^+^T cells via WB and qRT-PCR. (A) Representative immunoblots image (top) and quantification (bottom) of AARS1 in CD8^+^T cells. (B) The levels of *AARS1* in CD8^+^T cells measured by qRT-PCR. Data are presented as means ± SD, n = 3 independent experiments. **P* < 0.05, by one-way ANOVA followed by Bonferroni correction.

***Figure S13. Knockdown of AARS1 by siRNA reverses CSE-treated activation of CD8^+^T cells.***

AARS1 siRNA (50 µM) or NC siRNA (50 µM) was added to CD8⁺T cells 6 hours before 48-hour exposure to 0% or 4% CSE. (A) Representative immunoblot images (left) and quantitative analysis (right) of Pan-Kla in CD8^+^T cells. (B) Representative flow cytometry plots (top) and quantification (bottom) of IFN-γ^+^CD8^+^T cells. (C) Representative immunoblot images (left) and quantification (right) of Perforin, GZMB, and IFN-γ in CD8^+^T cells. (D) The levels of *Perforin*, *GZMB*, and *IFN-γ* in CD8^+^T cells measured by qRT-PCR. The levels of Perforin (E), GZMB (F), and IFN-γ (G) in the medium of CD8^+^T cells were quantified by ELISA. (H) Representative immunoblot images (left) and quantification (right) of Axin2, β-catenin, and Bcl-2/Bax in MLE-12 cells. Data are presented as means ± SD, n = 3 independent experiments. ***P* < 0.01, by one-way ANOVA followed by Bonferroni correction.

***Figure S14. Lactylation exists on the RUNX3 protein in CSE-treated CD8⁺T cells.***

(A) Co-immunoprecipitation (Co-IP) of RUNX3 and detection of Pan-kla by immunoblotting; (B) Reciprocal Co-IP of Pan-kla and detection of RUNX3 by immunoblotting.

***Figure S15. CSE elevates RUNX3 lactylation levels in CD8⁺T cells.***

(A) IP validation of RUNX3 acetylation in CD8⁺T cells with 0% or 4% CSE exposure. (B) IP validation of RUNX3 methylation in CD8⁺T cells with 0% or 4% CSE exposure. CD8^+^T cells were transfected with AARS1 overexpression plasmid (AARS1-OE) or empty vector (EV) for 6 hours. (C) The levels of AARS1 in CD8^+^T cells were measured by qRT-PCR. (D) IP validation of RUNX3 lactylation in CD8⁺T cells with AARS1-OE or EV. Data are presented as means ± SD, n = 3 independent experiments. ***P* < 0.01, by one-way ANOVA followed by Bonferroni correction.

***Figure S16. Downregulation of RUNX3 reverses CSE-treated CD8^+^T cell activation.***

Evaluation of RUNX3 knockdown efficiency by three siRNAs in CD8^+^T cells via WB and qRT-PCR. (A) Representative immunoblots image (top) and quantification (bottom) of RUNX3 in CD8^+^T cells. (B) The levels of *RUNX3* in CD8^+^T cells measured by qRT-PCR. RUNX3 siRNA (50 µM) or NC siRNA (50 µM) was added to CD8^+^T cells 6 hours before exposure to 0 % or 4 % CSE for 48 hours. (C) Representative immunoblot images (left) and quantification (right) of RUNX3 in CD8^+^T cells. (D) Representative flow cytometry plots (left) and quantification (right) of IFN-γ^+^CD8^+^T cells. (E) The levels of *Perforin*, *GZMB*, and *IFN-γ* in CD8^+^T cells measured by qRT-PCR. (F) Representative immunoblot images (left) and quantification (right) of Perforin, GZMB, and IFN-γ in CD8^+^T cells. The levels of Perforin (G), GZMB (H), and IFN-γ (I) in the medium of CD8^+^T cells were quantified by ELISA assay. (J) Representative immunoblot images (left) and quantification (right) of Axin2, β-catenin, and Bcl-2/Bax in MLE-12 cells. Data are presented as means ± SD, n = 3 independent experiments. **P* < 0.05, ***P* < 0.01, by one-way ANOVA followed by Bonferroni correction.

***Figure S17. RUNX3 binds to the promoter regions of Perforin, GZMB, and IFN-γ.***

CD8^+^T cells were exposed to 0% or 4% CSE for 48 hours. (A) The levels of RUNX3 in the Perforin promoter region of CD8^+^T cells as determined by ChIP-qPCR. (B) The levels of RUNX3 in the GZMB promoter region of CD8^+^T cells as determined by ChIP-qPCR. (C) The levels of RUNX3 in the IFN-γ promoter region of CD8^+^T cells as determined by ChIP-qPCR.

***Figure S18. Transfection efficiency of RUNX3 pcDNA in CD8^+^T cells.***

CD8^+^T cells were transfected with RUNX3 pcDNA or NC pcDNA for 6 hours. The levels of RUNX3 in CD8^+^T cells were measured by qRT-PCR. Data are presented as means ± SD, n = 3 independent experiments. ***P* < 0.01, by one-way ANOVA followed by Bonferroni correction.

***Figure S19. The elevation of serum lactate levels in CS-exposed mice.***

Six-week-old male BALB/c mice were exposed to CS for 24 weeks prior to measurement of serum lactate, with TPM concentrations in smoke set at 0, 200, or 300 mg/m³. Representative images (top) and quantification (bottom) of lactate in lung tissues. Data are presented as means ± SEM, n = 6 biological replicate. ***P* < 0.01, by one-way ANOVA followed by Bonferroni correction.

***Figure S20. Elevated human serum lactate is related to COPD progression.***

Correlative regression analysis was conducted to explore the relationship between human serum lactate levels in healthy participants (n=21, biological replicate) and COPD patients (n=15, biological replicate) with FEV1/FVC (%) or FEV1 predicted value (%).

***Figure S21. Elevated RUNX3 expression in pulmonary CD8⁺T cells correlates with COPD progression.***

(A) Representative RUNX3/CD8a co-localization immunostaining images (left) and corresponding quantitative analysis (right) of lungs from healthy donors (n=10, biological replicate) and COPD patients (n=10, biological replicate) (scale bar, 100μm). (B) Correlative regression analysis of RUNX3 expression with FEV1/FVC (%) or predicted FEV1 (%). Data are presented as means ± SEM. ***P* < 0.01, by Student’s unpaired t-test.

***Figure S22. Serum levels of AARS1 correlate positively with the serum levels of Perforin, GZMB, and IFN-γ in humans.***

Correlation regression analyses were performed to assess associations between

the serum levels of AARS1 and the serum levels of Perforin (A), GZMB (B), and IFN-γ (C), among healthy controls (n = 21, biological replicates) and patients diagnosed with COPD (n = 15, biological replicates).
